# CXCR4 coordinates adhesion, migration, and development of human NK cells

**DOI:** 10.1172/jci.insight.206701

**Published:** 2026-08-04

**Authors:** Shira E. Eisman, Francesca E. Grossberg, Batya S. Koenigsberg, David H. McDermott, Frédérique van den Haak, Luis A. Pedroza, Everardo Hegewisch-Solloa, Philip M. Murphy, Emily M. Mace

**Affiliations:** 1 Department of Pediatrics, Vagelos College of Physicians and Surgeons, and; 2 Columbia Stem Cell Initiative, Columbia University Irving Medical Center, New York, New York, USA.; 3 Laboratory of Molecular Immunology, National Institute of Allergy and Infectious Diseases, Bethesda, Maryland, USA.

**Keywords:** Cell biology, Immunology, Cell migration/adhesion, Chemokines, NK cells

## Abstract

Natural killer (NK) cells undergo stepwise differentiation from multipotent progenitors within secondary lymphoid tissues. Despite the central importance of the tissue microenvironment in their development, little is known about cell-cell interactions that regulate human NK cell trafficking and maturation. Here, we identify the chemokine receptor CXCR4 and its ligand CXCL12 as regulators of stromal–NK cell interactions required for NK cell maturation. We demonstrate that CXCR4 is expressed throughout human NK cell development in peripheral blood and tonsil, and CXCL12 is enriched in stromal niches containing developing NK cells. Pharmacologic blockade or genetic disruption of CXCR4 resulted in diminished adhesion to integrin ligands, and high-resolution imaging demonstrated crosstalk between CXCR4 and integrins, providing a mechanistic basis for chemokine-dependent modulation of adhesion. Further, CXCR4 blockade resulted in altered contact-dependent motility on stromal cells and integrin ligands, with decreased stable stromal engagement and increased cell speed. Consistent with a requirement for these interactions, treatment with the CXCR4 antagonist plerixafor (AMD3100) impaired NK cell generation from CD34^+^ precursors. Analysis of NK cells from patients with WHIM syndrome with CXCR4 gain-of-function mutations treated with plerixafor revealed similar defects in migration and adhesion, supporting the in vivo relevance of CXCR4-dependent regulation of NK cell adhesion and motility.

## Abstract 2

Eisman et al. show that the chemokine receptor CXCR4 regulates integrin-mediated tethering to stromal cells to promote human NK cell development and cell migration.

## Introduction

Natural killer (NK) cells are innate lymphocytes that contribute to early protection against viral infection and malignancy by exerting effector functions without the need for antigen presentation (1). Classically defined as CD3^–^CD56^+^ granular lymphocytes, NK cells originate from hematopoietic stem cells in the bone marrow and undergo stepwise maturation that can be defined by distinct surface markers and stage-specific functions (2, 3).

While recent studies show that early NK precursors leave the bone marrow and mature in secondary lymphoid tissues such as tonsil (4–7), the cellular interactions that guide human NK development remain incompletely understood. Fetal-liver-derived stromal cell lines support NK cell differentiation in vitro from CD34^+^ precursors (8–11), and direct stromal contact effectively promotes human NK maturation (12). While the molecular signals governing these interactions remain unclear, these observations point to a key role for stromal microenvironments that coordinate NK polarization, adhesion, and developmental progression. Previous studies of NK cells isolated from human peripheral blood show that they exhibit non-directed cell motility when plated on developmentally supportive stroma (13). NK cell subsets display distinct migratory behaviors on stromal cells, suggesting an intricate link between migration and development. A specialized NK-stroma contact termed the developmental synapse has been described and is marked by actin polarization, CD56 accumulation, and signaling activity (13). Together, these findings support a model in which regulated NK-stromal interactions contribute to human NK maturation, but the upstream cues that stabilize or modulate these contacts remain unknown.

Chemokine-receptor interactions likely stabilize these contacts. CXCR4 and its ligand CXCL12 regulate hematopoietic cell retention, homing, and tissue organization. Beyond their well-described role in chemotaxis (14, 15), CXCR4/CXCL12 can also promote synapse formation and polarization in developing immune cells (16). While CXCR4 has not been studied extensively in human NK cells, murine studies demonstrate its importance, as mice conditionally deficient in hematopoietic-lineage-specific CXCR4 display decreased NK precursors in the bone marrow and fewer mature NK cells in peripheral tissues (17). In addition, NK cells in mice localize near CXCL12-abundant reticular (CAR) cells that provide IL-15, an essential cytokine for NK development (18). These observations raise the possibility that CXCR4/CXCL12 actively shapes NK cell maturation through mechanisms beyond chemotaxis.

Aberrant leukocyte trafficking is a defining feature of primary immunodeficiencies that include NK cell abnormalities, including Wiskott-Aldrich syndrome and GATA2 deficiency (19, 20). Among disorders directly linked to dysregulated chemokine signaling, WHIM syndrome, a primary immunodeficiency defined by warts, hypogammaglobulinemia, infections, and myelokathexis, is the only currently recognized inborn error of immunity resulting from germline gain-of-function CXCR4 variants and results from gain-of-function mutations in *CXCR4* that most commonly truncate its C-terminal tail, impairing receptor internalization and causing sustained signaling (21–23). Hyperactive CXCR4 signaling enhances leukocyte retention in the bone marrow, resulting in pan-leukopenia, severe neutropenia, B cell lymphopenia with hypogammaglobulinemia, and recurrent infections, including human papilloma virus–associated (HPV-associated) warts and neoplasms (24–26). Despite this, many WHIM patients survive into adulthood, and long-term prognosis is usually influenced by chronic HPV-related disease rather than bacterial infections (26, 27). WHIM syndrome is exceedingly rare, with fewer than 200 cases reported worldwide to date (27). The onset of clinical symptoms is usually in childhood, although diagnosis is sometimes delayed to adulthood, with a median diagnosis at 5.5 years (27). Current treatments include immunoglobulin replacement therapy and antimicrobial prophylaxis as well as therapies targeting leukocyte mobilization. Granulocyte colony-stimulating factor (G-CSF; filgrastim) stimulates neutrophil production and indirectly promotes neutrophil egress by remodeling the bone marrow niche and indirectly suppressing stromal CXCL12. In contrast, CXCR4 antagonists such as plerixafor (AMD3100) directly block CXCR4 and rapidly mobilize neutrophils and multiple lymphocyte populations from the bone marrow. Clinical studies demonstrate that plerixafor produces infection outcomes comparable to those of filgrastim while also increasing absolute lymphocyte counts in peripheral blood and improving HPV-associated disease in WHIM syndrome patients (26, 28, 29).

Despite the central role of CXCR4 in NK cell biology, relatively little is known about NK cell dysfunction in WHIM syndrome or the effects of CXCR4 antagonism on NK cell biology. NK cell numbers are reduced in a subset of WHIM patients (30–32), and treatment with plerixafor leads to transiently increased NK cell numbers in the periphery of some patients (28). In mice, the expression of human WHIM syndrome variants causes increased bone marrow homing (17, 33). AMD3100 treatment in mice mobilizes NK cells to peripheral blood, and NK cell cytotoxic function is increased when bulk PBMCs are assayed (34); however, the effect of AMD3100 on the molecular function of NK cells has not been studied in depth (35). Together, these findings suggest that aberrant CXCR4 signaling influences NK cell trafficking, yet its effects on human NK cell migration, adhesion, development, and function remain poorly understood.

Here, we show that CXCR4 regulates human NK cell development by stabilizing integrin-dependent interactions with stromal cells. We demonstrate that CXCR4-CXCL12 interactions promote adhesion, polarization, and developmental progression of human NK cells, thus linking NK cell engagement with stromal cells to human NK cell maturation.

## Results

### CXCR4 is expressed on immature and mature human NK cells.

**Figure 1:**
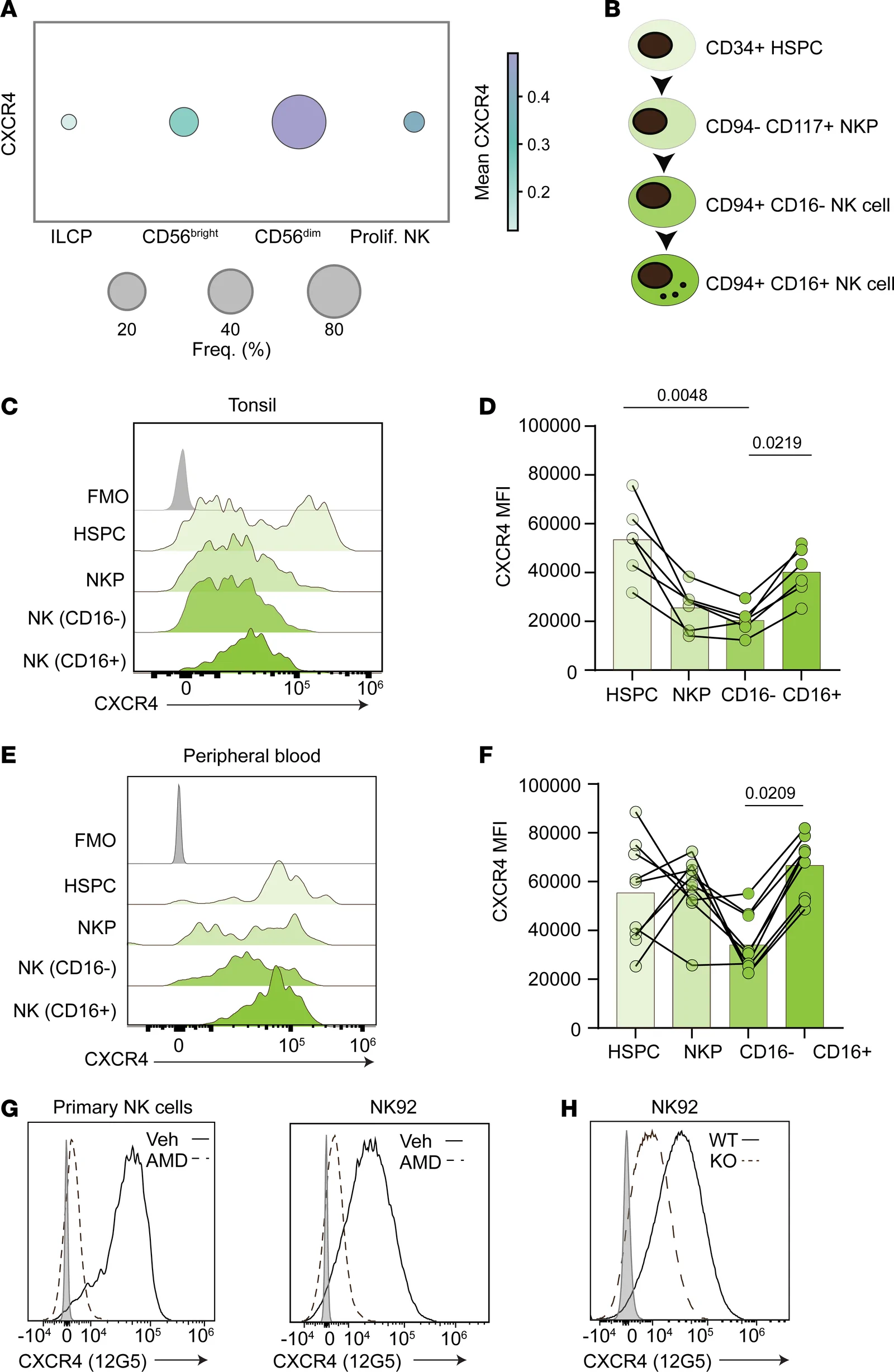
CXCR4 is expressed throughout human NK cell development. (**A**) CXCR4 transcript in innate lymphoid cell progenitors (ILCPs) and NK subsets from the Human Immune Health Atlas (66). Dot size indicates frequency of CXCR4^+^ cells; color represents mean expression. (**B**) NK cell development: CD34^+^ hematopoietic stem and progenitor cells (HSPCs), CD117^+^CD94^–^ NK precursors (NKPs), CD94^+^CD16^–^ NK cells, and CD94^+^CD16^+^ NK cells. (**C**) Extracellular CXCR4 in NK developmental intermediates from tonsil. Gray histogram represents fluorescence minus one (FMO) control. (**D**) Pooled data from 6 donors corresponding to flow cytometry data in **C**. *n* = 6; Friedman test with Dunn’s multiple-comparison correction (paired by donor). Only comparisons with *P* < 0.05 are displayed. (**E**) Extracellular CXCR4 mean fluorescence intensity (MFI) in NK developmental intermediates from peripheral blood. (**F**) Pooled data from 9 donors corresponding to flow cytometry data in **E**. *n* = 9; Friedman test with Dunn’s multiple-comparison correction (paired by donor). Only comparisons with *P* < 0.05 are displayed. (**G**) Representative histograms of cell surface CXCR4 MFI in primary NK cells (left) and NK92 cell lines (right) after 1-hour treatment with vehicle (solid lines) or 10 μM AMD3100 (dashed lines). Representative of more than 10 biological replicates. (**H**) Validation of CXCR4 knockout (KO) in NK92 cell line. CXCR4 on WT (solid line) and CRISPR/Cas9 CXCR4-KO (dashed line) cells. Representative of 4 replicates.

To investigate whether CXCR4/CXCL12 signaling might play a role in human NK cell development, we first assessed CXCR4 expression across NK cell developmental stages by examining CXCR4 transcript levels in circulating lymphoid progenitors and NK cell subsets using data from the Human Immune Health Atlas (36). *CXCR4* was detected in the innate lymphoid cell (ILC) progenitors detectable in PBMCs in this dataset, CD56^bright^ NK cells, CD56^dim^ NK cells, and proliferating NK cell subsets, with the highest mean expression in CD56^dim^ NK cells (Figure 1A).

We next assessed CXCR4 surface expression by flow cytometry on NK cell subsets and NK progenitors from tonsil, a site of human NK cell maturation (Figure 1B, table of antibodies in Supplemental Table 1, and gating strategy in Supplemental Figure 1A; supplemental material available online with this article; https://doi.org/10.1172/jci.insight.206701DS1). As predicted by its association with early hematopoietic differentiation (37, 38), we found significantly higher CXCR4 expression in CD34^+^ hematopoietic stem and progenitor cells (HSPCs) from tonsil when compared with mature CD16^–^ NK cells (Figure 1, C and D). Additionally, CD16^+^ NK cells from tonsil showed a significant increase in CXCR4 mean fluorescence intensity (MFI) relative to CD16^–^ NK cells (Figure 1, C and D), confirming that the higher expression of CXCR4 transcript in CD56^dim^ NK cells (Figure 1A) was also associated with higher protein expression. This higher protein expression seemed to be associated with NK cell developmental subsets, as CD16^–^ and CD16^+^ subsets of CD56^dim^ NK cells did not have significant differences in CXCR4 MFI (Supplemental Figure 1B). These data were supported by our analysis of scRNA-seq data from human bone marrow (39) showing similar levels of CXCR4 transcript in mature (CD56^+^) NK cell common lymphoid progenitors and other lineage-primed CD34^+^ subsets (Supplemental Figure 1C).

We then performed the same analysis using peripheral blood. Although CD34^+^ HSPCs are rare in peripheral blood, a similar trend toward higher expression in CD34^+^ HSPCs was observed relative to CD16^–^ NK cells (Figure 1, E and F). Circulating NK/ILC precursors (Lin^–^CD117^+^CD94^–^) in peripheral blood (40) showed comparable CXCR4 expression to HSPCs. As in tonsil, CD16^+^ NK cells displayed higher CXCR4 MFI than CD16^–^ NK cell subsets (Figure 1, E and F). Together, these flow cytometry data demonstrated that CXCR4 is expressed at all stages of human NK cell development in peripheral blood and tonsil, with differences in expression associated with changes in NK cell maturation and location.

To establish experimental models for CXCR4 perturbation, we used pharmacologic blockade with a CXCR4 antagonist (AMD3100) on primary NK cells and generated a CRISPR/Cas9–mediated CXCR4 knockout (KO) in the NK92 NK cell line. As expected, AMD3100 treatment of primary NK cells and NK92 cells reduced CXCR4 detection by flow cytometry as a result of competitive binding with the CXCR4-specific antibody clone 12G5 (Figure 1G) (41, 42). Targeted knockout of CXCR4 using CRISPR/Cas9 resulted in a single population with approximately 50% decreased CXCR4 protein expression by flow cytometry (Figure 1H). Neither pharmacologic blockade nor genetic deletion affected NK cell viability or proliferation (Supplemental Figure 2A). In Transwell assays, both AMD3100 and CXCR4 knockout significantly impaired directed migration toward CXCL12, confirming that both approaches impacted the canonical function of CXCR4 in chemotaxis (Supplemental Figure 2B). Decreased chemotaxis in the CXCR4-KO cells was moderate but could be further reduced in the presence of AMD3100 (Supplemental Figure 2B). In contrast, NK cell cytotoxic function in a standard 4-hour killing assay against K562 target cells was only modestly reduced by CXCR4 knockout or AMD3100 (Supplemental Figure 2C). Together, these results demonstrate that CXCR4 is expressed throughout NK cell development and in NK cell lines, and that it is not required for NK cell survival or cytotoxic function.

### CXCR4 ligand CXCL12 is found in NK cell developmental niches.

CD34^+^ progenitors in peripheral blood that give rise to NK cells are thought to enter the tonsil through high endothelial venules (HEVs) within the interfollicular domain, then migrate to the parafollicular domain, where they undergo further maturation (3). As CXCL12 is the primary ligand for CXCR4, we sought to define its spatial localization in tonsil using cyclic immunofluorescence (CycIF) on formalin-fixed, paraffin-embedded sections from routine pediatric tonsillectomies (43).

**Figure 2:**
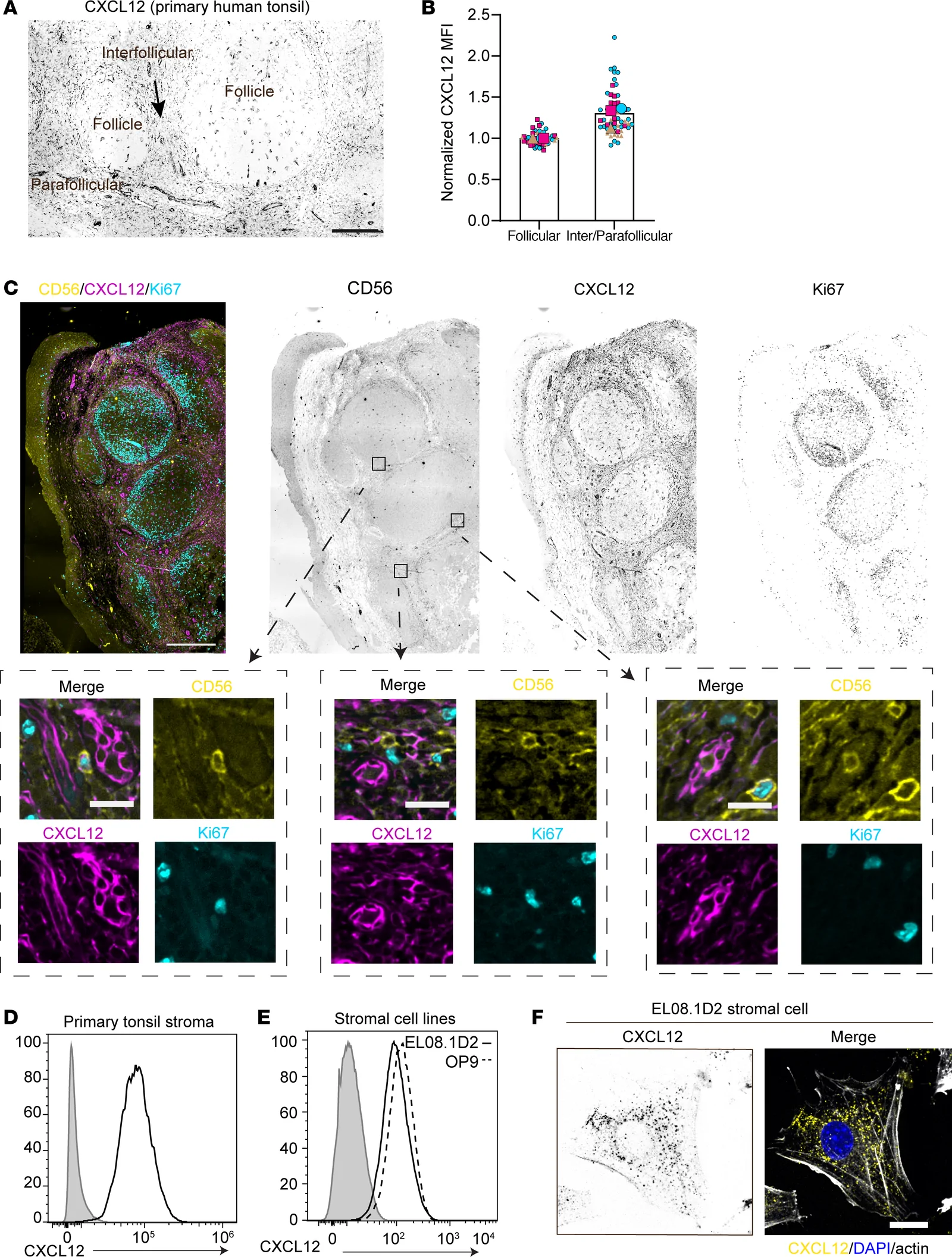
CXCL12 is found in sites that support human NK cell development. Cyclic immunofluorescence was performed on pediatric human tonsil. (**A**) Representative immunofluorescence staining of CXCL12 in primary human tonsil tissue with follicles and interfollicular and parafollicular regions annotated. Scale bar: 200 μm. (**B**) Quantification of normalized CXCL12 MFI comparing follicular versus para- and interfollicular regions; each point represents a region of interest from 3 donors, 8–12 regions per donor. Large symbols represent mean of values from individual donors. (**C**) Cyclic immunofluorescence staining of pediatric tonsil showing CD56^+^ NK cells (yellow), CXCL12 (magenta), and Ki67 (cyan). Grayscale images show individual channels CD56, CXCL12, and Ki67. Bottom panels show higher-magnification views of NK cell–rich para- and interfollicular stromal regions with CD56^+^ NK cells adjacent to CXCL12^+^ stroma. Scale bars: 25 μm. (**D**) Flow cytometric analysis of primary tonsil stromal cells stained for intracellular CXCL12 (white) compared with fluorescence minus one (FMO) control (gray). (**E**) CXCL12 expression by stromal cell lines. Flow cytometry comparing intracellular CXCL12 expression on EL08.1D2 (solid line) versus OP9 (dashed line) stromal cells with FMO control (gray). (**F**) Confocal imaging of EL08.1D2 stromal cells showing CXCL12 (yellow), DAPI (blue), and actin (white). Scale bar: 5 μm.

Observing CXCL12 expression in CycIF images, we found that CXCL12 protein was distributed throughout the tonsil but enriched in inter- and parafollicular stromal regions surrounding follicles and in HEVs (Figure 2A). Quantification of multiple regions of interest from samples from multiple donors confirmed higher CXCL12 intensity in inter- and parafollicular regions relative to B cell follicles (Figure 2B). Using our multiplexed images, we defined CD56^+^CD3^−^ NK cells and used Ki67 to visualize proliferating cells (Figure 2C; additional channels shown in Supplemental Figure 3). High-magnification images showed that NK cells were often adjacent to CXCL12^+^ stromal cells; and many, but not all, of these were Ki67^+^ (Figure 2C). Further analysis revealed that CD34^+^ progenitors were also found adjacent to CXCL12^+^ stroma (Supplemental Figure 2B). These data were supported by recent studies using multiplexed immunofluorescence that showed that proliferative NK cell progenitors were significantly enriched proximal to CXCL12^+^ cells (43).

To further demonstrate that tonsil stromal cells are a source of CXCL12, we isolated CD45^−^ primary tonsil stromal cells from routine tonsillectomies. Flow cytometry demonstrated that primary tonsil stroma cells uniformly expressed CXCL12 that was detected following fixation and permeabilization (Figure 2D). We also found that cell lines that support NK cell development, including OP9 and EL08.1D2, similarly expressed CXCL12 (Figure 2E). We confirmed this finding with confocal microscopy of EL08.1D2, which showed intracellular and cell surface expression of CXCL12 (Figure 2F). These results are consistent with CXCL12 being produced intracellularly for secretion and subsequent binding to the extracellular matrix (44, 45). Together, these data demonstrate that CXCL12 is produced by stromal cells in the inter- and parafollicular zones, forming chemokine-rich microenvironments that likely guide NK progenitors and other CXCR4^+^ cells in the tonsil (43).

### CXCR4 promotes integrin-mediated interactions between NK cells and stroma.

**Figure 3:**
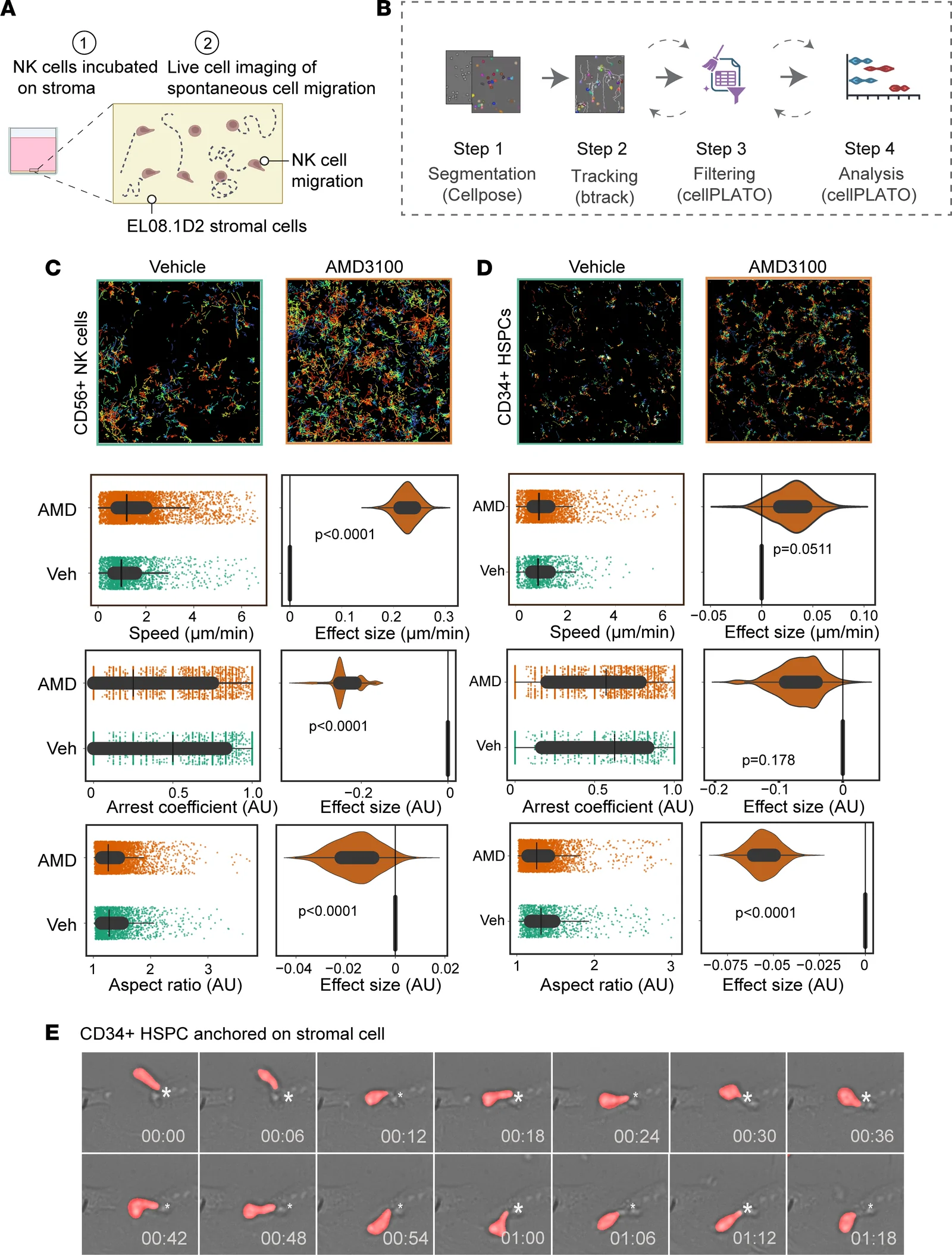
CXCR4-CXCL12 interaction promotes integrin-mediated arrest on stromal cells. Primary CD56^+^ NK cells and CD34^+^ progenitors were plated on EL08.1D2 stromal monolayers or integrin ligand surfaces and imaged by confocal microscopy every 2 minutes for 2–6 hours. (**A**) Schematic of NK cell migration assay. (**B**) Overview of the cellPLATO analysis pipeline. Time-lapse images were segmented using Cellpose, tracked using btrack, and processed through the cellPLATO workflow. (**C**) Migration of primary NK cells treated with 10 μM AMD3100 or vehicle on stroma. Top: Representative segmentation masks and cell trajectories. Scale bar: 100 μm. Color gradient signifies time. Plots of differences show mean speed, arrest coefficient, and aspect ratio. Each point represents a single cell. Effect size was calculated by bootstrap resampling. (**D**) Migration of CD34^+^ progenitor cells treated with 10 μM AMD3100 or vehicle on stroma. Top: Representative segmented images and cell tracks of migrating CD34^+^ cells. Scale bar: 100 μm. Color gradient signifies time. Migration and shape parameters for CD34^+^ cells, including mean speed, arrest coefficient, and aspect ratio, are shown. (**E**) Time-lapse sequence of CD34^+^ HSPC with segmentation shown in red interacting with a stromal cell. Asterisk shows the point of contact. Time is shown as hh:mm. See also Supplemental Video 2 and Supplemental Table 2.

Since NK cell development depends on direct contact with stromal cells and NK cells undergo surveillance-like migration on stromal monolayers in vitro (12, 13), we asked whether CXCR4/CXCL12 contributes to these migratory behaviors. CD56^+^ NK cells and CD34^+^ progenitors from cord blood were pretreated with vehicle (water) or AMD3100 and plated on EL08.1D2 stromal monolayers. To assess these interactions in the context of spontaneous cell migration, cells were imaged every 2 minutes for 2–6 hours by live-cell confocal microscopy (Figure 3A). We used Cellpose (46) to segment the cells, btrack (47) to track the cells, and Cell Plasticity Analysis Tool (cellPLATO) (48) to quantify and plot cell motility and shape parameters (Figure 3B). This analysis focuses on spontaneous contact-mediated motility, in contrast to the directed chemotactic migration measured in Transwell assays (Supplemental Figure 2B).

We first analyzed the migration of primary NK cells (Figure 3C). Individual cell trajectories over time were visibly longer and less confined in AMD3100-treated cultures (Figure 3C and Supplemental Videos 1 and 2), suggesting changes in migratory behavior resulting from CXCR4 inhibition. We quantified cell migration parameters, including cell speed and arrest coefficient (the frequency of time a cell is immotile as defined by moving under 1 μm/min). To visualize these differences, we used plots of differences (49) to display the effect size distribution of more than 8,000 cells between conditions. In the vehicle-treated condition, NK cells exhibited characteristic non-directed migration on stromal monolayers, with heterogeneous motility patterns and intermittent arrest (Supplemental Video 1). Overall, AMD3100 treatment increased motility and reduced arrest on stroma (Figure 3C and Supplemental Video 2). Specifically, AMD3100-treated cells had significantly higher average speeds (1.233 μm/min vs. 0.986 μm/min for vehicle) with a reduction in arrest coefficient (0.374 vs. 0.483 for vehicle). These data reflect the AMD3100-treated NK cells becoming more motile and less adherent to the stromal surface. Additionally, shape analysis demonstrated rounder, less elongated cells under AMD3100 treatment (aspect ratio of 1.312 vs. 1.319 for vehicle). Similar trends were observed in additional biological replicates (Supplemental Figure 4 and Supplemental Table 2).

Next, given the expression of CXCR4 on CD34^+^ HSPCs (Figure 1), we asked whether AMD3100 treatment similarly affects CD34^+^ progenitor cell migration and engagement with stroma (Figure 3D and Supplemental Videos 3 and 4). Although these cells were overall less motile than mature NK cells (13, 50), AMD3100 similarly increased cell speed and modestly reduced arrest coefficient, with rounder and less polarized cells. These data indicate that CXCR4 contributes to progenitor-stroma interactions as well. Consistent with these population-level differences, time-lapse imaging showed cells in the CD34^+^ vehicle-treated condition that remained stably anchored to individual stromal cells for more than 1 hour, with ongoing protrusive activity but little translocation (Figure 3E and Supplemental Video 5), a phenotype that has been previously described for HSPCs in contact with bone marrow stroma (16) and was reduced upon AMD3100 treatment (Supplemental Video 6).

To confirm that these observations were not unique to primary cells or pharmacologic blocking of CXCR4, we next analyzed migration of CXCR4-wild-type (WT) and CXCR4-KO NK92 cell lines. Like primary NK cells treated with AMD3100, CXCR4 knockout increased cell speed while decreasing arrest coefficient (Supplemental Figure 4B, Supplemental Videos 7 and 8, and Supplemental Table 2).

Since NK cell migration and adhesion are known to be mediated by integrin-ligand interactions (51), we next assessed the migration of primary NK cells with and without AMD3100 on integrin ligands ICAM-1 or VCAM-1 (Supplemental Videos 9–12). In both cases, AMD3100 treatment similarly increased motility and reduced arrest, suggesting that CXCR4 signaling directly reinforces integrin-dependent contact with both stromal cells and integrin ligands (Supplemental Figure 4C and Supplemental Table 2). Altogether, these findings indicate that CXCR4 contributes to integrin-dependent adhesion and motility.

### CXCR4 promotes integrin activation and NK cell adhesion.

**Figure 4:**
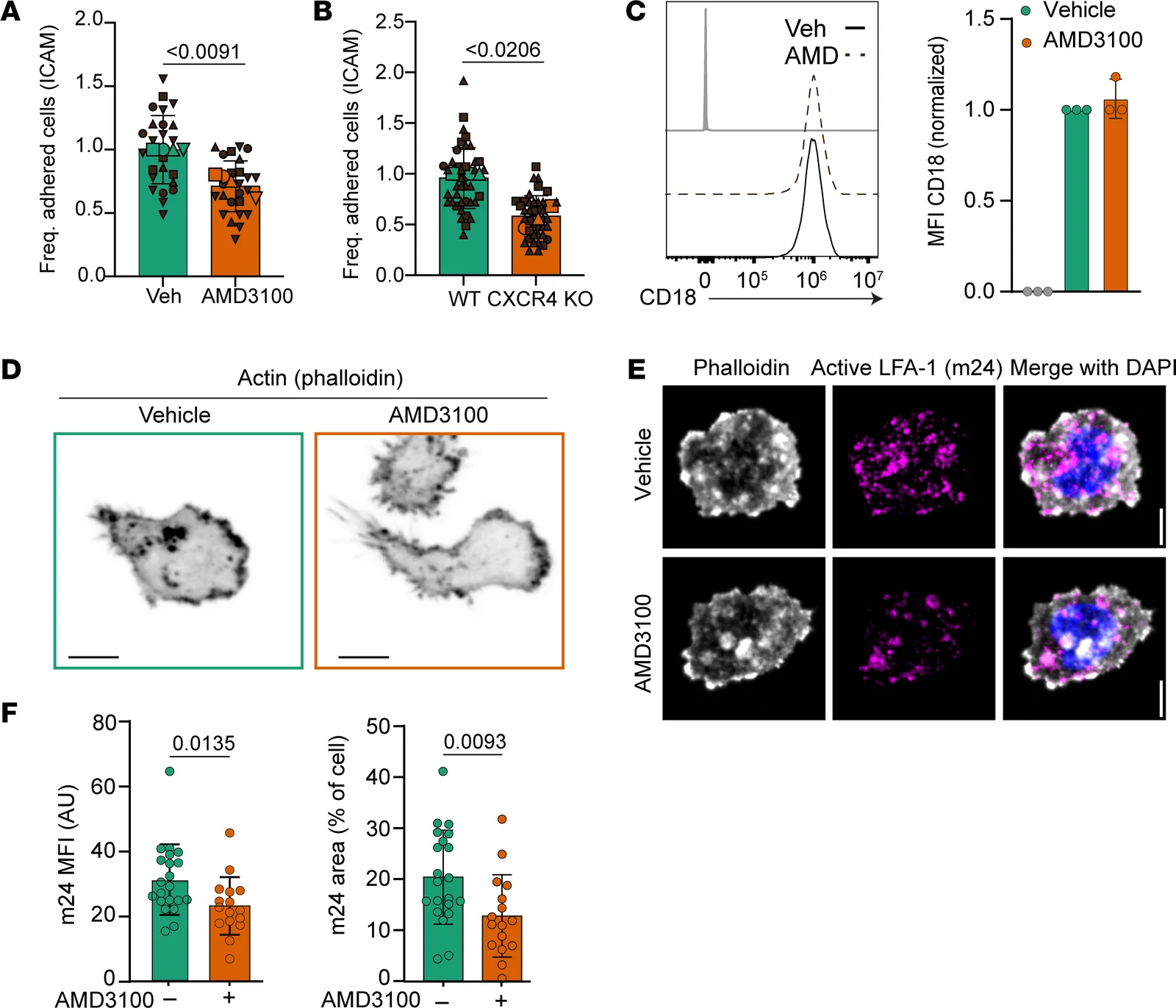
CXCR4 signaling promotes adhesion and integrin activation. (**A**) Cell adhesion assays of primary CD56^+^ NK cells on ICAM-1 following vehicle or 10 μM AMD3100 treatment. *n* = 4 donors; mean of each vehicle-treated donor is normalized to 1. (**B**) Adhesion of NK92 WT or CXCR4-KO cells to ICAM-1–coated plates. *n* = 3 independent experiments; large symbols represent the mean of each technical replicate. Statistical analysis by 2-tailed 1-sample *t* test compared with a theoretical mean of 1. (**C**) Surface CD18 expression in primary NK cells after treatment with vehicle (water) or AMD3100 displayed as representative histograms (left) and normalized MFI (right). Gray, fluorescence minus one (FMO) control. *n* = 3 independent experiments (mean ± SD). No statistical significance by 2-tailed Wilcoxon’s matched-pairs signed-rank test. (**D**) Representative confocal imaging of actin in NK92 cells treated with 10 μM of AMD3100 or vehicle and incubated on ICAM-1, then fixed and immunostained with phalloidin. Scale bars: 5 μm. Data are representative of 3 independent experiments/donors. (**E**) Primary CD56^+^ NK cells were treated with 10 μM of AMD3100 or vehicle and incubated on ICAM-1 (LFA-1 ligand) before fixation and immunostaining with anti–activated LFA-1 (clone m24), phalloidin, and DAPI. Scale bars: 5 μm; maximum projection shown. (**F**) Quantification of m24 MFI and percentage area from one representative donor. Each dot represents a single cell from one donor. Horizontal bars indicate mean ± SD. Statistical comparison by 2-tailed Mann-Whitney test across single-cell measurements within this donor. Data are representative of 3 independent experiments/donors (see also Supplemental Figure 5).

To directly test whether CXCR4 modulates NK cell adhesion via integrin activation, we measured adhesion to ICAM-1. Pharmacologic inhibition of CXCR4 significantly reduced the adhesion of primary NK cells to ICAM-1–coated surfaces, and similar reductions were observed in CXCR4-KO NK92 cells, indicating that CXCR4 activity enhances ICAM-1 engagement (Figure 4, A and B). Despite the decrease in adhesion, surface expression of CD18 was unchanged (Figure 4C), indicating that reduced adhesion is not due to changes in integrin surface abundance. We also found no obvious differences in cell morphology or actin architecture after inhibition of CXCR4 function (Figure 4D).

We therefore examined integrin activation on primary CD56^+^ NK cells using the conformation-sensitive antibody m24, which recognizes the high-affinity state of LFA-1 (52). CXCR4 inhibition with AMD3100 markedly reduced m24 intensity and cluster formation on the NK cell surface following ICAM-1 engagement (Figure 4E). Quantification of these observations confirmed that AMD3100 significantly decreased the size and intensity of m24 foci (Figure 4F). Two additional donors are included in Supplemental Figure 5. Together, these findings demonstrate that CXCR4 signaling promotes β_2_ integrin activation and ICAM-1–dependent adhesion. This establishes a mechanistic link between CXCR4 and the stabilization of NK cell–stroma interactions, positioning CXCR4 as an upstream regulator of the adhesion-migration balance in human NK cells.

### CXCR4 and CXCL12 are required for effective NK cell differentiation from CD34^+^ cells.

**Figure 5:**
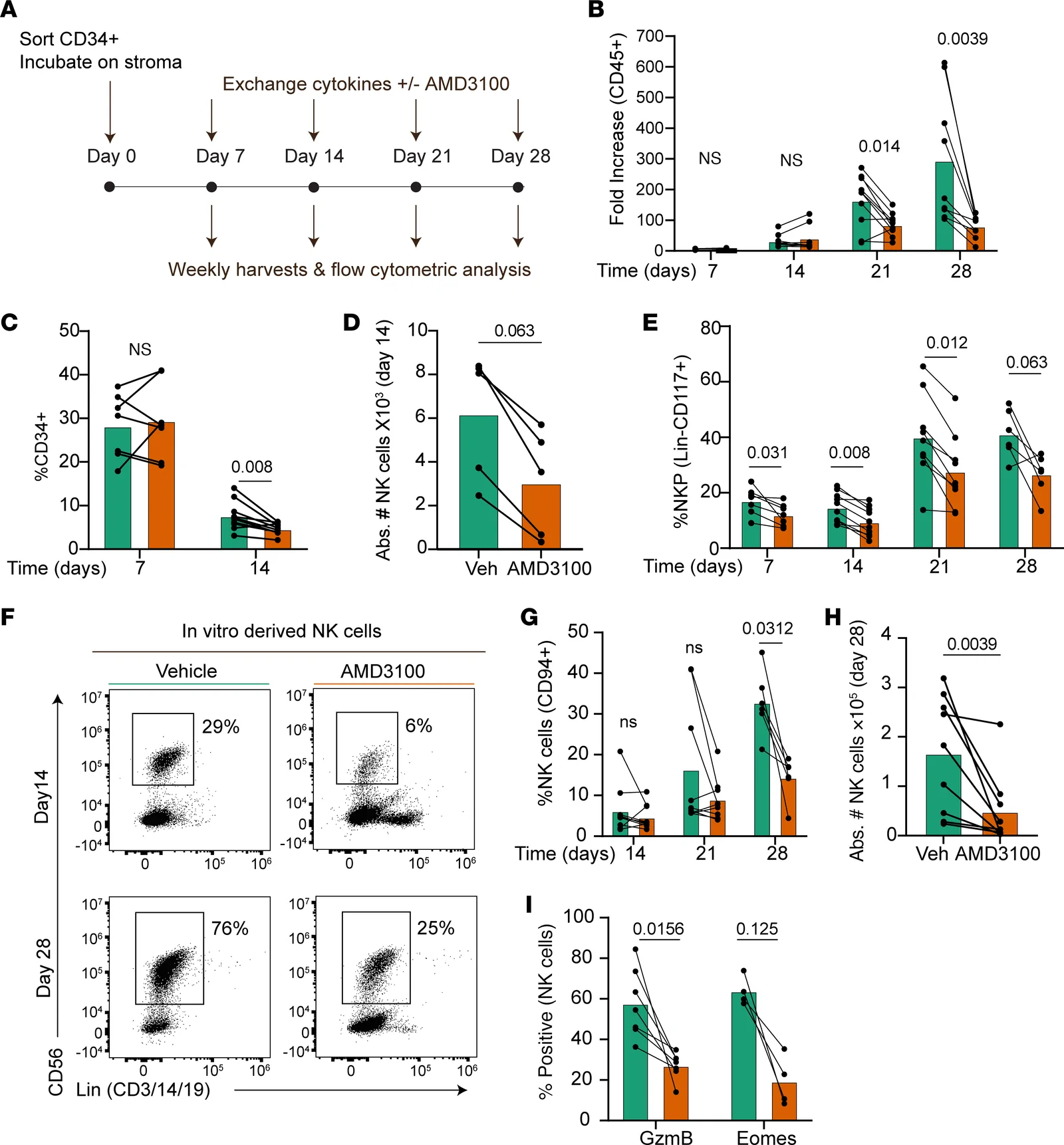
CXCR4-CXCL12 interactions are required for effective NK cell differentiation from CD34^+^ cells. (**A**) Schematic of NK cell in vitro differentiation protocol. Human CD34^+^ cells were isolated from cord blood using density centrifugation and fluorescence-activated cell sorting (FACS). Sorted CD34^+^ cells were plated on EL08.1D2 stromal cells with cytokines as indicated. Cultures were treated with 10 μM AMD3100 or vehicle at weekly media exchanges. (**B**) Fold increase in the absolute number of live CD45^+^ cells from AMD3100-treated (orange) and vehicle-treated (green) cultures. *n* = 4–10 donors. (**C**) Frequency of CD34^+^ cells gated on CD45^+^ cells at days 7 and 14. *n* = 7–10 donors. (**D**) Absolute number (×10^3^) of NK cells at day 14. *n* = 5 donors. (**E**) Frequency of Lin^–^CD117^+^ NK/ILC progenitors gated on CD45^+^ live cells by week. *n* = 7–9 donors. For **B**–**E**, each dot represents an individual donor, and lines connect paired samples. Statistical comparisons between vehicle and AMD3100 conditions at each time point were performed using Wilcoxon’s matched-pairs signed-rank tests. (**F**) Representative flow plots from days 14 and 28 showing frequencies of CD56^+^ Lin^–^ NK cells. Previously gated on CD45^+^ live cells. (**G**) Frequencies of CD94^+^ conventional NK cells at days 14, 21, and 28. *n* = 7–9 donors. (**H**) Absolute number (×10^5^) of CD94^+^ conventional NK cells at day 28. *n* = 9 donors. (**I**) Frequencies of granzyme B^+^ (GzmB) and EOMES^+^ NK cells at day 28. *n* = 4–7 donors.

Because stromal contact is required for human NK cell maturation, we next asked whether disruption of CXCR4-dependent adhesion affects NK cell developmental progression. To test this, we performed NK cell differentiation from HSPCs in the presence or absence of AMD3100. CD34^+^ cord blood progenitors were isolated by FACS and differentiated on EL08.1D2 stromal monolayers with cytokines (8). Cultures were treated with 10 μM AMD3100 or vehicle (water) at weekly media exchanges (Figure 5A). At weekly harvests, we quantified total live CD45^+^ cells and NK developmental subsets by flow cytometry.

We first examined the fold increase of live CD45^+^ cells in each culture condition over time (Figure 5B). Vehicle- and AMD3100-treated cultures were not significantly different through day 14. However, by day 21, a clear divergence emerged whereby vehicle-treated cultures underwent a robust expansion and AMD3100-treated cultures failed to expand. This indicates that CXCR4 signaling becomes particularly important during later stages of in vitro NK cell differentiation when cells normally proliferate in response to stromal cues.

To test whether reduced output reflected impaired hematopoietic progenitor maintenance or differentiation, we measured CD34^+^ frequencies at days 7 and 14 and found no significant difference between conditions at day 7 (Figure 5C), suggesting that early differentiation is not stalled in the presence of AMD3100. As expected, the frequency of CD34^+^ cells decreased from about 30% to under 10% between weeks 1 and 2 in both groups, consistent with CD34 downregulation during progression through differentiation, and at day 14 we found significantly fewer CD34^+^ progenitors in the AMD3100-treated condition (Figure 5C) (10). This progression through differentiation was accompanied by the appearance of NK cells in these cultures, with a decrease in the absolute number of NK cells at 14 days noted in the AMD3100 condition (Figure 5D). Next, we looked at CD117^+^ Lin^–^ NK/ILC precursors (40), a substantial population of cells in in vitro differentiation cultures (8, 9). AMD3100 cultures contained significantly fewer NK precursors (NKPs) than vehicle controls at multiple time points when measured by frequency (Figure 5E). This reduction in intermediate precursors suggests that CXCR4 signaling supports the progression and/or maintenance of developing NK cells beyond early progenitor stages. Representative flow cytometry plots illustrate the robust accumulation of CD56^+^ NK cells in vehicle-treated cultures compared with the reduced population in AMD3100-treated cultures (Figure 5F). The effect of AMD3100 on other, non-NK cell populations in these cultures cannot be ruled out; however, the decreased absolute number of NKPs and mature NK cells in our cultures precludes the different frequency of these cells being a result of altered frequencies of other subsets.

By day 28, AMD3100-treated cultures showed a clear reduction in CD94^+^CD56^+^ mature NK cells (Figure 5G). This observation was consistent with the failure of AMD3100 cultures to undergo the late expansion observed in vehicle-treated cultures. In addition to frequency, the absolute number of NK cells generated at 28 days was also significantly lower for AMD3100 cultures in comparison with vehicle (Figure 5H). NK cells generated in AMD3100 cultures also showed significantly reduced frequency of granzyme B^+^ cells at day 28, with a consistent reduction in EOMES^+^ cells across donors that did not reach statistical significance (paired Wilcoxon’s test) (Figure 5I). These markers reflect acquisition of effector functions and transcriptional maturation, indicating that CXCR4 blockade affects multiple markers of NK cell maturation and/or homeostasis. Whether there are effects on non-NK cells, particularly myeloid cells and other ILC subsets, that are generated in concert with conventional NK cells remains to be determined.

In summary, AMD3100 treatment during in vitro differentiation limits the generation and maturation of NK cells from CD34^+^ progenitors by reducing NK/ILC precursors and committed NK output and decreasing cytotoxic and transcriptional maturation markers. These findings suggest that CXCR4/CXCL12 signaling provides critical, stage-specific developmental cues, likely through niche positioning and stromal contact interactions, that support efficient human NK cell development.

### Plerixafor treatment of WHIM syndrome patients alters NK cell motility, mirroring in vitro AMD3100 effects.

**Figure 6:**
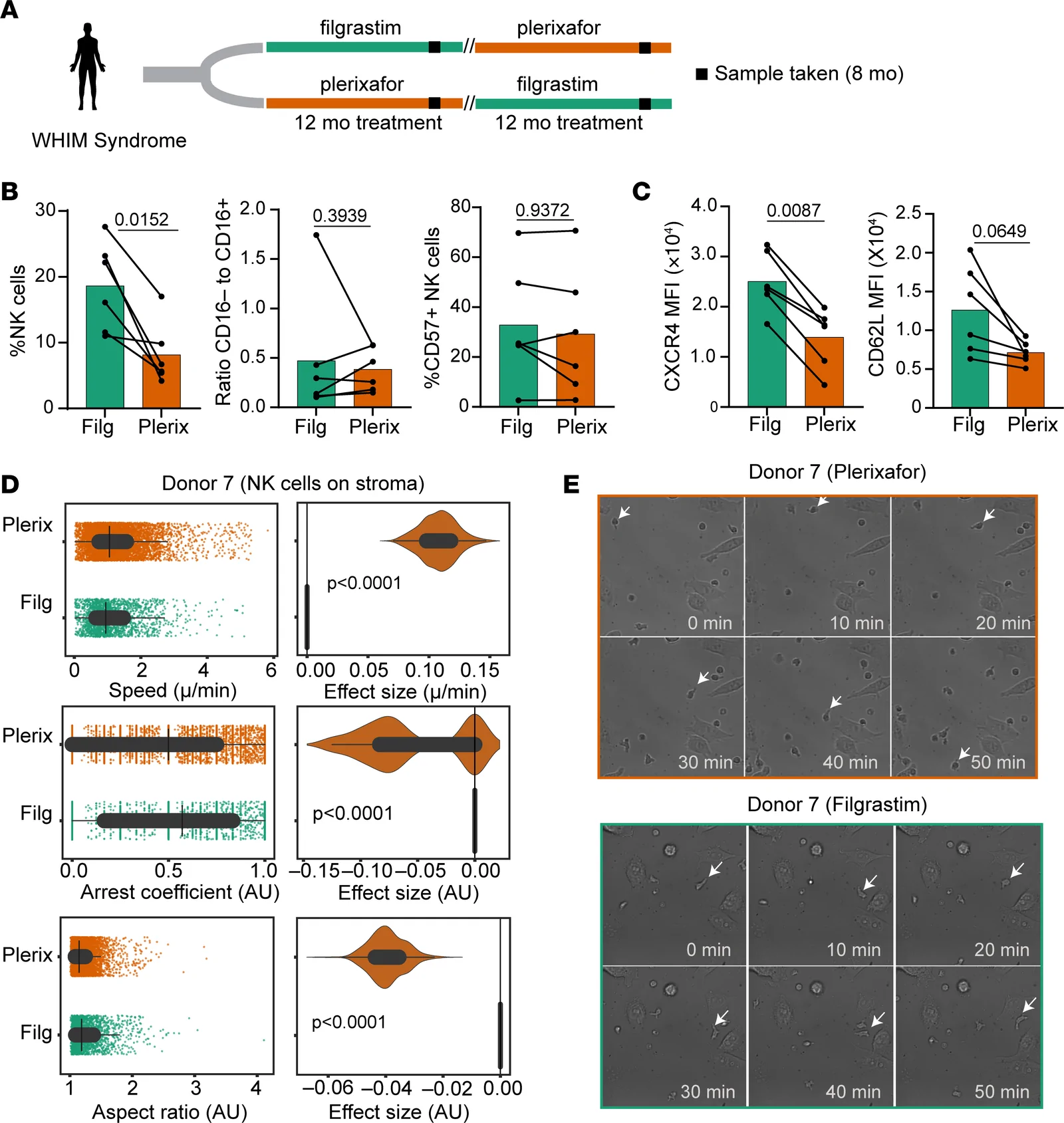
Plerixafor treatment of WHIM syndrome patients alters NK cell phenotype and motility. (**A**) WHIM samples were obtained after 8 months of treatment following either filgrastim or plerixafor administration as previously described (29). (**B**) NK cell frequency and CD16^+^ and CD57^+^ subsets in paired samples from the same individual undergoing filgrastim (Filg) or plerixafor (Plerix) treatments. *n* = 6 donors; mean ± SD; Wilcoxon’s matched-pairs signed-rank tests. (**C**) Surface CXCR4 and CD62L expression on NK cells from paired samples from the same individual undergoing filgrastim or plerixafor treatments. *n* = 6; mean ± SD; Mann-Whitney test. (**D**) NK cells were isolated from peripheral blood of WHIM syndrome individuals undergoing plerixafor or filgrastim treatment. Cells were imaged on EL08.1D2 stromal cells for 4 hours at 2-minute intervals. Motility and shape parameters comparing NK cells of treated WHIM patients. Data were paired between post-filgrastim and post-plerixafor samples from 12 WHIM donors. Point plots display pooled single-cell metrics with effect size distributions. (**E**) Representative time-lapse imaging of post-plerixafor and post-filgrastim treated NK cells (white arrows) on stromal cells.

Because our in vitro data suggest that CXCR4 blockade alters NK cell phenotypes, we asked whether these features are mirrored ex vivo in samples from WHIM patients with gain-of-function CXCR4 mutations undergoing treatment (28). To characterize NK cell phenotype in WHIM syndrome, we analyzed peripheral blood from healthy donors (HDs) and WHIM patients by flow cytometry. All WHIM samples were collected after 8 months of treatment in a 12-month course of filgrastim (G-CSF) or the CXCR4 antagonist plerixafor (AMD3100) (Figure 6A). Filgrastim mobilizes CXCR4^+^ cells by altering the CXCL12 in the stromal niche, while plerixafor directly blocks CXCR4-CXCL12 interactions (28). Since paired samples were available, we compared phenotypes during filgrastim versus plerixafor treatment from the same individual. Pretreatment samples were not available.

WHIM syndrome patients undergoing treatment with filgrastim or plerixafor collectively displayed a significantly increased percentage of NK cells among live CD45^+^ cells from peripheral blood compared with HDs, with skewing toward the less mature CD16^–^ population (Supplemental Figure 6). Surface CXCR4 expression was modestly lower on NK cells from treated WHIM patients compared with HDs (Supplemental Figure 6). Given that CXCR4 gain-of-function mutations in WHIM are classically associated with impaired receptor internalization and heightened CXCL12 responsiveness, this is surprising. However, this reduction in detection likely reflects the residual binding of plerixafor to CXCR4, which is known to compete for antibody binding with detection antibody. We also found that CD62L expression was significantly reduced in WHIM patient NK cells (Supplemental Figure 6), suggesting additional mechanisms of altered maturation and/or homing of NK cells in individuals with WHIM syndrome undergoing treatment.

To analyze treatment-specific effects, we compared paired samples during filgrastim or plerixafor treatment from individual WHIM donors. The frequency of CD56^+^ NK cells among CD45^+^ leukocytes was lower after plerixafor treatment compared with filgrastim (Figure 6B). This is expected, as more lymphocyte populations are released into circulation in plerixafor-treated versus filgrastim-treated patients (28). Moreover, CXCR4 surface levels were lower in plerixafor-treated NK cells relative to filgrastim-treated samples (Figure 6C), consistent with plerixafor occupancy blocking the 12G5 antibody epitope. CD62L expression also trended lower under plerixafor treatment, though this difference did not reach significance.

To assess how treatment influences NK cell migratory behavior, CD56^+^ NK cells were sorted from patient samples and plated on EL08.1D2 stromal monolayers. NK cells from filgrastim- versus plerixafor-treated WHIM patients displayed striking differences in morphology and motility (Figure 6, D and E, and Supplemental Table 2). NK cells from filgrastim-treated patients showed more elongated and polarized morphologies, whereas NK cells from plerixafor-treated patients were markedly rounded. Additionally, NK cells from plerixafor-treated patients exhibited lower aspect ratio, reduced elongation, and increased rounding (Figure 6D), indicating impaired polarization. They also showed significantly lower arrest coefficient, suggesting reduced stable substrate engagement. Instantaneous speeds were modestly higher in NK cells from plerixafor-treated patients, consistent with a non-adherent, exploratory mode of movement. Examples from time-lapse imaging confirmed these differences, as filgrastim-treated NK cells formed persistent leading edges and stable contacts with stroma, whereas plerixafor-treated NK cells remained spherical, transiently contacting the surface but failing to establish durable adhesions (Figure 6E). This phenotype closely mirrors the reduced adhesiveness and polarization we observed with AMD3100 (plerixafor) treatment in vitro, reinforcing that CXCR4 blockade directly disrupts adhesion and cytoskeletal organization.

Taken together, these findings indicate that pharmacologic CXCR4 antagonism disrupts polarization and stable substrate engagement of NK cells, potentially impairing their ability to navigate tissues or form long-lived immune synapses. While plerixafor is clinically effective for leukocyte mobilization and WHIM management, our results suggest that transient impairment of NK cell adhesion and polarity may represent a short-term tradeoff of CXCR4 blockade, with potential implications for NK surveillance and effector functions during treatment.

## Discussion

Our data support a model in which CXCR4-CXCL12 interactions orchestrate NK cell positioning and adhesion within developmental niches, thus enabling efficient NK cell maturation. CXCL12 is highly expressed on tonsil stromal cells, particularly on fibroblastic reticular cells (53, 54), and is abundant in para- and interfollicular regions that also support developing NK cells (43). In the thymus, CXCL12 is crucial for positioning T cells for effective T cell development and proliferation (55, 56), and in the bone marrow, CXCL12-associated reticular (CAR) cell niches are sites of NK cell development (17). As fibroblastic reticular cells in lymph nodes can also produce IL-15 (57), which is necessary for NK cell maturation, we propose that CXCL12 could play a role in recruiting and retaining HSPCs and NKPs in sites that are enriched with cytokines that promote NK cell differentiation. This model is analogous to the role of CAR cells in murine bone marrow (17) and suggests that CXCR4/CXCL12 signaling integrates tissue localization with access to differentiation cues. The specific localization of NK progenitors to endothelial cells expressing CXCL12 and Flt3L in tonsil strongly suggests that there are niches in tonsil that provide necessary and sufficient signaling for human NK cell development (43).

Beyond positioning NK cells within CXCL12-rich niches, our data indicate that CXCR4 plays a key role in balancing NK cell motility and adhesion. Pharmacologic blockade of CXCR4 results in cells that are more motile, less arrested, and rounder on developmentally supportive stromal cells. Consistent with reduced adhesion, CXCR4 inhibition impairs β_2_ integrin activation, suggesting that CXCR4 stabilizes cell contacts. CXCR4-dependent adhesive structures have been described in other hematopoietic lineages; HSPCs form highly polarized, CXCR4/CXCL12-dependent “magnupodia” that anchor them to bone marrow stromal cells (16, 58). Developing T cells at the β-selection checkpoint likewise assemble a CXCR4-dependent immunological synapse with thymic stroma that is required for progression through this developmental checkpoint (59, 60). This CXCR4-dependent shift in cells from an adhesive, elongated migration phenotype to a rounded, high-motility state demonstrates how NK cells can integrate environmental cues to transition between migratory and adherent behaviors. Although this balance is classically described in the context of a cytotoxic synapse (61), a similar principle likely applies in non-cytolytic developmental settings. CXCR4/CXCL12 signaling provides positional information that coordinates adhesion, integrin activation, and motility to regulate how NK cells engage with stromal cells and extracellular matrix. While the molecular mechanisms underscoring these changes in cell behavior remain unclear, they do not appear to result from gross defects in actin organization, as AMD3100-treated cells exhibit no obvious alterations in cell morphology or actin architecture (Figure 4).

The functional consequences of this altered adhesion are evident in our differentiation system, where AMD3100-treated cultures show reduced generation of mature NK cells and incomplete cytotoxic maturation. These observations are consistent with prior work showing that NK differentiation on EL08.1D2 stroma is contact dependent (11). In addition, our previous description of the developmental synapse demonstrated that developing NK cells polarize and form stable CD56- and actin-rich contacts with stromal cells that are required for efficient human NK cell differentiation (13). These findings suggest that reduced stable interactions with stromal cells under CXCR4 blockade prevent progenitors from establishing polarity or stable signaling contacts with stroma that are required for full NK lineage commitment. Interestingly, this altered adhesion does not significantly negatively affect target cell killing, as we found only mild impairment in the killing of K562 cells by AMD3100-treated or CXCR4-KO NK92 cells. It is likely that residual integrin expression is sufficient to preserve cytotoxic function at the synapse, particularly in the context of highly susceptible target cells. This finding is supported by the observation that NK cells from individuals with leukocyte adhesion deficiency type 1, resulting from loss-of-function mutations in LFA-1, retain their capacity to kill tumor target cell lines (62). While limited access to patient material prevented us from performing assays to measure NK cell cytotoxic function, it would be interesting to test in future studies whether the tuning of LFA-1 function by CXCR4 manipulation would lead to changes in NK cell function, particularly in a solid tumor model requiring cell adhesion and migration or in other tumor types aside from K562 cells.

The relevance of our findings is underscored by the analysis of samples from patients with WHIM syndrome, a disorder driven by hyperactive CXCR4 signaling (25). Although CXCR4 dysfunction in WHIM syndrome has been well studied in neutrophils and B cells, its impact on NK cells has been less clear, with prior reports describing either reduced or unchanged NK cell frequencies in peripheral blood (29, 30). In our cohort, NK cells collected during plerixafor treatment exhibit a rounded morphology and reduced arrest behavior, closely mirroring the phenotype of NK cells treated with AMD3100 in vitro. Detection of surface CXCR4 and CD62L was also reduced during plerixafor treatment, consistent with CXCR4 receptor occupancy and altered trafficking. These findings suggest that while pharmacologic CXCR4 antagonism is effective for hematopoietic mobilization, it may transiently impair the ability of NK cells to form polarized, adhesive contacts with substrates. It is important to note that filgrastim and plerixafor mobilize leukocytes through distinct mechanisms, namely a G-CSF–mediated remodeling of the stromal niches versus direct CXCR4 antagonism (30, 63), which likely contributes to the treatment-dependent differences in NK behavior we observed. Additionally, as expected, differences between in vitro and ex vivo phenotypes likely reflect distinct drug exposure dynamics.

Several limitations should be considered in interpreting these findings. First, our WHIM cohort size was limited, and untreated baseline samples were not available, preventing direct assessment of the intrinsic NK phenotype in WHIM syndrome. Second, this study overall did not examine potential crosstalk between CXCR4 and other receptors, including S1P5, which is known to influence NK cell egress (64) and may intersect with CXCR4-dependent positioning. Third, although EL08.1D2 stroma recreate key features of CXCL12-rich niches, in vivo developmental contacts likely involve additional stromal subsets and extracellular matrix components not modeled here. Fourth, we did not dissect in depth the potential functional differences between mature or circulating NK cell subsets and those that spend more time in tissue, such as CD56^bright^ NK cells. Nonetheless, the convergent in vivo and in vitro observations support a model in which CXCR4 signaling regulates NK cell adhesion and motility in human physiology. Supporting this broader role, disruptions of the CXCR4/CXCL12 axis in tumor models enhance NK cell infiltration (65), also mirroring the increased migration we observe under CXCR4 blockade.

Together, these data identify CXCR4/CXCL12 as a key regulator of the balance between migration, adhesion, and maturation in human NK cells. Our findings place CXCR4/CXCL12 at the center of a regulatory circuit that coordinates NK cell adhesion, motility, and developmental progression. By tuning integrin activation and stabilizing interactions with stromal niches, CXCR4 helps ensure that developing NK cells receive the signals necessary for efficient maturation.

## Methods

### Sex as a biological variable.

Sex was not analyzed as a biological variable in this study. Human peripheral blood and umbilical cord blood samples were obtained from deidentified donors, and donor sex information was not provided. WHIM samples were used from 5 female donors and 1 male donor, consistent with a previously described sex bias in WHIM syndrome (27, 35). Samples were included without sex-based stratification, and analyses were focused on CXCR4/CXCL12-dependent cellular behaviors across donors rather than sex-specific differences.

### Study design.

Experiments were performed using primary human NK cells and progenitor populations isolated from healthy donor peripheral blood from adults over 18 years of age, cord blood, and tonsil tissue from pediatric donors, as well as established human NK cell lines. PBMCs from WHIM patients treated with filgrastim or plerixafor were analyzed in select experiments. These samples were shared from a clinical trial of patients who were 12–57 years of age (28). Depending on the assay, experimental units consisted of independent donor samples, cell cultures, or individual cells. Peripheral blood and tonsil cells were not paired, and WHIM patient samples were not age and sex matched. Measurements included flow cytometry, quantitative imaging, and live-cell migration analysis. Sample sizes were determined based on sample availability and prior experience with similar assays. Biological replicates represent independent donors or independent cell culture experiments, and technical replicates represent repeated measurements within the same experiment. Data inclusion criteria were defined before analysis. Samples were included if viability and assay quality controls met predefined thresholds. Live-cell imaging datasets were analyzed using automated segmentation and cell-tracking pipelines, and tracks were filtered using predefined criteria (including minimum track duration and segmentation quality). Data were excluded only in cases of technical failure (poor staining, low viability, or imaging artifacts). Outliers were identified using the ROUT method (*Q* = 1%) in GraphPad Prism when applicable. Statistical tests used for each analysis are described in the figure legends. Data acquisition and computational analyses were applied uniformly across experimental conditions.

### Public dataset analysis.

CXCR4 expression data for circulating lymphoid progenitors and NK cell subsets were obtained from the Human Immune Health Atlas (66). For each annotated cluster, we calculated the mean CXCR4 transcript levels and the proportion of CXCR4-expressing cells. Bubble plots were generated in Python using Scanpy for data processing and Matplotlib to visualize average expression (color scale) and frequency of expressing cells (bubble size). CXCR4 expression of bone marrow cells was calculated from scRNA-seq data downloaded from Hay et al. (39).

### Sample processing.

Peripheral blood was obtained from healthy donors via venipuncture at Columbia University Medical Center. Tonsil samples were acquired from routine tonsillectomies on pediatric patients at Columbia University Medical Center. Tonsil tissue was mechanically dissociated by mincing with sterile scalpels and passing of the suspension through a 40 μm cell strainer (Falcon, 352340). Cell suspensions were washed with PBS by centrifugation at 300*g* for 10 minutes at room temperature. For flow cytometric analysis, cells were resuspended in PBS and stained with relevant antibodies or resuspended in FBS 10% DMSO freezing medium and cryopreserved.

Deidentified PBMCs were isolated from the blood of participants in a clinical trial of the treatment of WHIM patients with plerixafor versus filgrastim (28) (ClinicalTrials.gov NCT02231879). This was a single-center, investigator-initiated, blinded crossover trial in which each participant received each treatment in succession with a randomization of which drug started first. Cryopreserved cells from participants were deidentified and used in this study. Both drugs were administered twice daily subcutaneously using prefilled syringes, and matched samples used here were drawn at the 6-month time point of the 1-year treatment period (8 months of drug exposure counting a 2-month initial dose adjustment period). Untreated baseline samples were not available from these donors.

Deidentified umbilical cord blood samples were acquired from the placenta of women who were undergoing routine healthy term deliveries (between 37 weeks 0 days and 41 weeks 6 days of gestation). Blood was collected in either BD Vacutainer with sodium heparin or BD Vacutainer with 7.2 mg K2 EDTA. Collection followed institutional standard operating procedures and was performed in accordance with the AIDS Clinical Trials Group and IMPAACT Network Umbilical Cord Blood Collection Standard Operating Procedure (67).

Mononuclear cells were isolated from whole blood and cord blood by Ficoll-Paque (Cytiva, catalog 17144003) density gradient centrifugation. NK cells and progenitor cells were enriched using RosetteSep negative selection enrichment kits (NK cell enrichment, STEMCELL Technologies, catalog 15065; hematopoietic stem cell enrichment, STEMCELL Technologies, catalog 15066) following manufacturer instructions.

For in vitro differentiation experiments, CD34^+^ precursors were isolated from cord blood samples. Mononuclear cells isolated from donors were incubated with anti-CD34 antibody (BioLegend, clone 581), and CD34^+^ cells were isolated by FACS on a Sony MA900 sorter.

### Cell culture.

NK92 cells (ATCC) were maintained in complete NK92 growth medium consisting of α-MEM without ribonucleosides or deoxyribonucleosides (Gibco, 12-561-056) supplemented with 12.5% heat-inactivated horse serum, 12.5% heat-inactivated FBS, 2 mM l-glutamine, 1.5 g/L sodium bicarbonate, 0.2 mM myo-inositol, 0.1 mM β-mercaptoethanol, 0.02 mM folic acid, and 100 U/mL recombinant human IL-2. Medium was filtered before use. NK92 cells were maintained at 37°C with 5% CO_2_ and passaged every 2–3 days.

EL08.1D2 stromal cells (provided by E.A. Dzierzak, Erasmus MC, Netherlands, via J.S. Miller, University of Minnesota) were cultured on gelatin-coated plates at 32°C in medium composed of 40.5% α-MEM (Life Technologies), 50% Myelocult (STEMCELL Technologies), 7.5% heat-inactivated FBS supplemented with 2 mM GlutaMAX, 100 U/mL penicillin/streptomycin, 50 mM β-mercaptoethanol, and 10^–6^ M hydrocortisone. For coculture experiments, EL08.1D2 cells were plated into gelatinized 96-well plates and mitotically inactivated by irradiation at 3 Gy before the addition of progenitor cells.

OP9 cells were a gift from A.G. Freud (The Ohio State University, Columbus, Ohio, USA) and were cultured in α-MEM with 20% heat-inactivated FBS on tissue culture plates precoated with 0.1% gelatin. K562 cells were a gift from J.S. Orange (Children’s Hospital of Philadelphia, Philadelphia, Pennsylvania, USA) and were maintained in RPMI 10% heat-inactivated FBS.

### Flow cytometry.

Freshly isolated or cryopreserved cells were thawed into warm R10 medium and then washed with PBS. Cryopreserved cells were allowed to recover in R10 medium at 37°C for 3 hours before staining or sorting. Cells were stained for extracellular markers for 30 minutes at 4°C and then washed with PBS 1% BSA. When intracellular markers were stained, cells were fixed and permeabilized using eBioscience FoxP3 fixation/permeabilization buffer (Thermo Fisher Scientific, catalog 00-5523-00), then stained for intracellular markers for 30 minutes at 4°C followed by washes with FoxP3 wash buffer. Data were acquired on a Novocyte Penteon Cell Analyzer and then exported to FlowJo (BD Biosciences) for analysis. A list of antibodies is given in Supplemental Table 1.

### Transwell migration assay.

Transwell migration assays were performed using Corning Transwell permeable supports with a 6.5 mm insert, 8.0 μm pores in a 24-well plate (catalog 3422), or CELLTREAT permeable cell culture inserts in a 12-well plate, 8.0 μm pores (catalog 230619). For each condition, 600 μL Opti-MEM medium (Thermo Fisher Scientific) supplemented with CXCL12 (200 ng/mL) and/or AMD3100 (10 μM) was added to the lower chamber. Recombinant IL-2 (100 U/mL) was included for NK92 cells. Cells were pelleted and resuspended in Opti-MEM containing IL-2 with or without AMD3100, and 5 × 10^5^ cells in 100 μL were added to the upper chamber. Assays were performed in duplicate. Plates were incubated for 5 hours at 37°C, and migrated cells were collected by rinsing of the underside of each insert with 400 μL PBS. Cells in the lower well were counted.

### CRISPR/Cas9 gene editing.

Two million NK92 cells were nucleofected with 5 μM of sgRNA guide specific for CXCR4 (Horizon, sgRNA CXCR4 [ATGCTGATCCCAATGTAGTA], catalog SG-005139-01-0002) and 5 μg of Cas9-GFP mRNA (Horizon) in 100 μL of Nucleofector Kit-R supplemented solution (Lonza). The R-024 program of an Amaxa Nucleofector II (Lonza) was used. After 24 hours, GFP^+^ cells were sorted and cultured before validation.

### Cytotoxicity assay.

Four-hour Cr^51^ release assays were performed using K562 target cells as described previously (68). Target cells were used at a concentration of 10^4^ target cells per well of a 96-well U-bottom plate. Effector cells were used in serial dilution starting at an effector-to-target-cell ratio of 50:1 for PBMCs or 10:1 for NK92 cells. After incubation, supernatants were transferred into Lumaplates (Revvity) and measured using a TopCountXL (PerkinElmer). Specific lysis (percentage) was calculated as (experimental cpm – spontaneously released cpm)/(total cpm – spontaneously released cpm) × 100.

### Cyclic immunofluorescence and analysis of formalin-fixed, paraffin-embedded pediatric tonsil sections.

Formalin-fixed, paraffin-embedded tonsil sections were stored at –20°C and brought to room temperature before processing. After three 10-minute xylene (Fisher Scientific, catalog X5-1) washes, tissue was rehydrated by sequential incubations in 100% and 95% ethanol (Fisher Scientific, catalog BP2818100) and deionized water for 10 minutes, and then in 70% and 35% ethanol and deionized water for 5 minutes. For heat-activated antigen retrieval, slides were incubated in pre-warmed Agilent Dako pH 6 antigen retrieval solution (Agilent, catalog S2367) for 20 minutes in a vegetable steamer (Black & Decker), cooled to room temperature, and washed with PBS.

Tissue sections were incubated with 1 mg/mL borohydride in deionized water for 10 minutes to decrease background fluorescence, washed 3 times with PBS, permeabilized with 0.2% Triton X-100 in PBS for 30 minutes, and washed with PBS 3 times. Next, a hydrophobic border was drawn with a peroxidase-antiperoxidase pen, and tissue samples were incubated with SuperBlock Blocking Buffer (Thermo Fisher Scientific, catalog 37580) for 1 hour.

Antibodies for immunostaining (Supplemental Table 1) were diluted in an antibody diluent buffer (PBS/10% BSA/0.01% Triton X-100). Immunostaining of tissue sections with unconjugated antibodies for high-resolution immunofluorescence microscopy was done overnight at 4°C in a humidity chamber. Slides were washed with antibody diluent buffer 4 times after immunostaining. Tissue sections were incubated with secondary antibodies at a dilution of 1:200 for 1 hour at room temperature followed by 2 washes with antibody diluent buffer and 5 washes with PBS. PBS was removed, and then tissue was treated with a Vector TrueVIEW Autofluorescence Quenching Kit (Vector Laboratories Inc., catalog SP-8400) before mounting. After washing of excess TrueVIEW reagents 3 times with PBS, tissue sections were mounted using VectaShield Antifade Mounting Medium with DAPI (Vector Laboratories Inc., catalog H-1200) and a #1.5 coverslip (Electron Microscopy Services, catalog 72204-03). Regions of interest were imaged with a 20% overlap using a Zeiss CellDiscoverer 7 with a ×20/0.7 or ×20/0.95 NA objective and an optical magnification of ×0.5, and coordinates were saved for subsequent imaging. This resulted in images with a microns-per-pixel unit of 0.454. After imaging, coverslips were gently removed by washing with PBS in Coplin jars.

To strip antibodies, tissue slides were incubated in freshly prepared stripping buffer (20 mL 10% SDS, 0.8 mL β-mercaptoethanol, 12.5 mL 0.5 M Tris-HCl [pH 6.8], 67.5 mL deionized water) for 40 minutes in a vegetable steamer, then washed with water for 15 minutes. Slides were then blocked with SuperBlock for 10 minutes and restained with primary and secondary antibodies before reimaging as described above. Tile stitching, channel registration, and barrel-distortion correction were performed using ASHLAR (69). All downstream visualization was performed in Fiji (70).

### Proliferation assays.

NK92 WT and NK92 CXCR4-KO cells were cultured in complete NK92 medium with 1,000 U/mL recombinant human IL-2 at 37°C and 5% CO_2_. Cells were seeded at 2 × 10^5^ cells/mL in 24-well plates and treated with AMD3100 (10 μM) or vehicle control (water). Live-cell concentrations were measured at 0, 24, 48, and 72 hours using acridine orange/propidium iodide staining and an automated cell counter (Cellometer; Nexcelom).

### Confocal imaging and analysis.

For fixed-cell imaging of stromal cells, EL08.1D2 stromal cells were cultured on gelatin-coated chamber slides (ibidi) until 70%–80% confluent. Cells were fixed with 4% paraformaldehyde for 10 minutes at room temperature, washed with PBS, and permeabilized with 0.1% Triton X-100 for 10 minutes. After blocking with 5% BSA in PBS for 1 hour, cells were incubated with anti-CXCL12 conjugated antibody (clone 79018, R&D Systems, FAB350T; 1:100) and phalloidin (Thermo Fisher Scientific, A22287; 1:200) for 1 hour at room temperature. Slides were washed 3 times in PBS, followed by DAPI nuclear staining.

Primary NK cells were washed and incubated in HBSS with Ca^2+^Mg^2+^ for 30 minutes at 37°C. AMD3100 (10 μM) was added for 30 minutes where indicated. Cells were stimulated with CXCL12 (200 ng/mL) for 5 minutes and immediately fixed in 4% paraformaldehyde for 10 minutes. After washing and permeabilizing with 0.1% Triton X-100, cells were stained with the anti–LFA-1 Alexa Fluor 488 antibody (clone m24, BioLegend, 363404), phalloidin Alexa Fluor 568 (Thermo Fisher Scientific, A12380), and DAPI (Millipore, D9542).

Confocal images were acquired on a Yokogawa spinning-disk confocal microscope on a Zeiss Axioimager Z1 stand with a ×63 1.4 NA objective using matched laser and exposure settings. Images were processed and analyzed using SlideBook (Intelligent Imaging Innovations) and Fiji (70). *Z*-stacks (0.3 to 0.5 μm steps) were collected, and maximum-intensity projections were generated in Fiji. Cell segmentation was performed using actin masks, and m24 MFI and m24^+^ membrane area measurements were extracted for each cell. At least 10 cells per condition per donor were analyzed.

### Live-cell migration imaging and analysis.

Primary NK cells, CD34^+^ progenitors, or NK cell lines were pretreated with vehicle control (water) or AMD3100 (10 μM) for 2 hours at 37°C. For stromal cocultures, EL08.1D2 stromal cells were plated as confluent monolayers 24 hours before imaging. For integrin ligand surfaces, wells were coated overnight at 4°C with 5 μg/mL recombinant human ICAM-1–Fc (Sino Biological, 10346-H02H) or VCAM-1–Fc (BioLegend, 553706), followed by 2 PBS washes. Cells were added gently to the monolayer or ligand surface in NK medium. Live-cell imaging was performed on a spinning-disk confocal microscope equipped with a 37°C 5% CO_2_ environmental chamber. Bright-field images were acquired every 2 minutes for 2–6 hours using a ×20 objective on a Yokogawa spinning-disk confocal microscope on a Zeiss Axioimager Z1 stand with a ×63 1.4 NA objective. Time-lapse movies were exported as TIFF stacks for analysis.

Segmentation of individual cells was performed using Cellpose (v2) (46). Segmentation masks were visually inspected for accuracy. Cell tracking was performed using btrack (47). Tracks shorter than 2 frames were removed. Tracking results were visually reviewed in napari to confirm accuracy. Segmentation and tracking outputs were filtered, as described for each experiment.

Quantitative migration metrics were computed using cellPLATO as described by Shannon et al. (48), including cumulative track length, instantaneous speed, and arrest coefficient (fraction of frames in which displacement was less than 1 μm/min). Morphological features (cell area, aspect ratio, and solidity) were calculated from segmentation masks on a per-frame basis and averaged for each cell.

Migration and shape metrics were analyzed at the single-cell level. Differences between treatment conditions were evaluated using bootstrap resampling and permutation-based *P* values. Effect size and confidence intervals were visualized using plots of differences. Statistical analyses were performed using custom Python scripts.

### In vitro NK cell differentiation on EL08.1D2 stromal cells.

In vitro NK cell differentiation was performed as previously reported (8). Human CD34^+^ hematopoietic progenitors were isolated from cord blood by FACS sorting, and 400 cells per well were plated onto an irradiated EL08.1D2 stromal cell monolayer. Differentiation cultures were maintained in Ham’s F12 medium plus DMEM (1:2) with 20% human AB serum, ethanolamine (50 μM), ascorbic acid (20 μg/mL), sodium selenite (5 μg/L), β-mercaptoethanol (24 μM), and penicillin/streptomycin (100 U/mL) with IL-15 (5 ng/mL), IL-3 (5 ng/mL), IL-7 (200 ng/mL), SCF (20 ng/mL), and Flt3L (10 ng/mL). Half the volume of medium was exchanged weekly. For AMD3100 conditions, 10 μM AMD3100 was added to cultures with weekly media exchanges. Developing NK cells were collected at indicated time points for flow cytometry analysis.

### Statistics.

Statistical analyses were performed with Prism 10.0.0 (GraphPad). Unpaired samples were measured with parametric (2-tailed *t* test) or non-parametric (Mann-Whitney) tests depending on whether data fell within a normal distribution. Paired data were compared using Wilcoxon’s paired signed-rank test. Paired comparisons across NK developmental populations were analyzed using a Friedman test with Dunn’s correction for multiple comparisons. Each donor was treated as an independent biological replicate. Outliers were identified using the ROUT method (*Q* = 1%) in GraphPad Prism.

### Study approval.

All human samples were collected in accordance with the Declaration of Helsinki and approved by the Columbia University Institutional Review Board (IRB) or NIH IRB. Informed consent was obtained from all donors or their legal guardians prior to sample collection, and all samples were deidentified before analysis.

### Data availability.

All data supporting the findings of this study are included in the article and its supplemental materials. A Supporting Data values XLS file is also included. Additional datasets and analysis outputs generated during the current study are available upon reasonable request.

## Author contributions

SEE performed experiments, analyzed data, and wrote the manuscript. EMM conceived and designed the study, provided conceptual guidance, supervised the study, and wrote the manuscript. FG, FVDH, LAP, and BSK performed experiments. EHS contributed technical expertise and guidance. DHM and PMM contributed patient samples and WHIM-specific expertise. All authors reviewed and approved the final manuscript.

## Conflict of interest

The authors have declared that no conflict of interest exists.

## Funding support

This work is the result of NIH funding, in whole or in part, and is subject to the NIH Public Access Policy. Through acceptance of this federal funding, the NIH has been given a right to make the work publicly available in PubMed Central.

- NIH T32GM007088 to SEE and R01AI137073 to EMM.
- The work on WHIM patient samples was partially funded by the Division of Intramural and Clinical Research of the National Institute of Allergy and Infectious Diseases.
- Molecular Pathology Shared Resource of the Herbert Irving Comprehensive Cancer Center at Columbia University Irving Medical Center, which is funded through the National Cancer Institute Cancer Center Support Grant (P30CA013696).

## Supplementary Material

Supplemental data

Supplemental video 1

Supplemental video 10

Supplemental video 11

Supplemental video 12

Supplemental video 2

Supplemental video 3

Supplemental video 4

Supplemental video 5

Supplemental video 6

Supplemental video 7

Supplemental video 8

Supplemental video 9

Supporting data values

## Version 1 08/04/2026

In-Press Preview

## Version 2 09/08/2026

Electronic publication
