# Supplementary material for: CXCR4 coordinates adhesion, migration, and development of human NK cells
Source: JCI Insight. 2026 Aug 4;11(17):e206701. doi: 10.1172/jci.insight.206701 (PMC13564093; doi:10.1172/jci.insight.206701)
Supplement: Supplementary file 1 [file jciinsight-11-206701-s171.pdf]

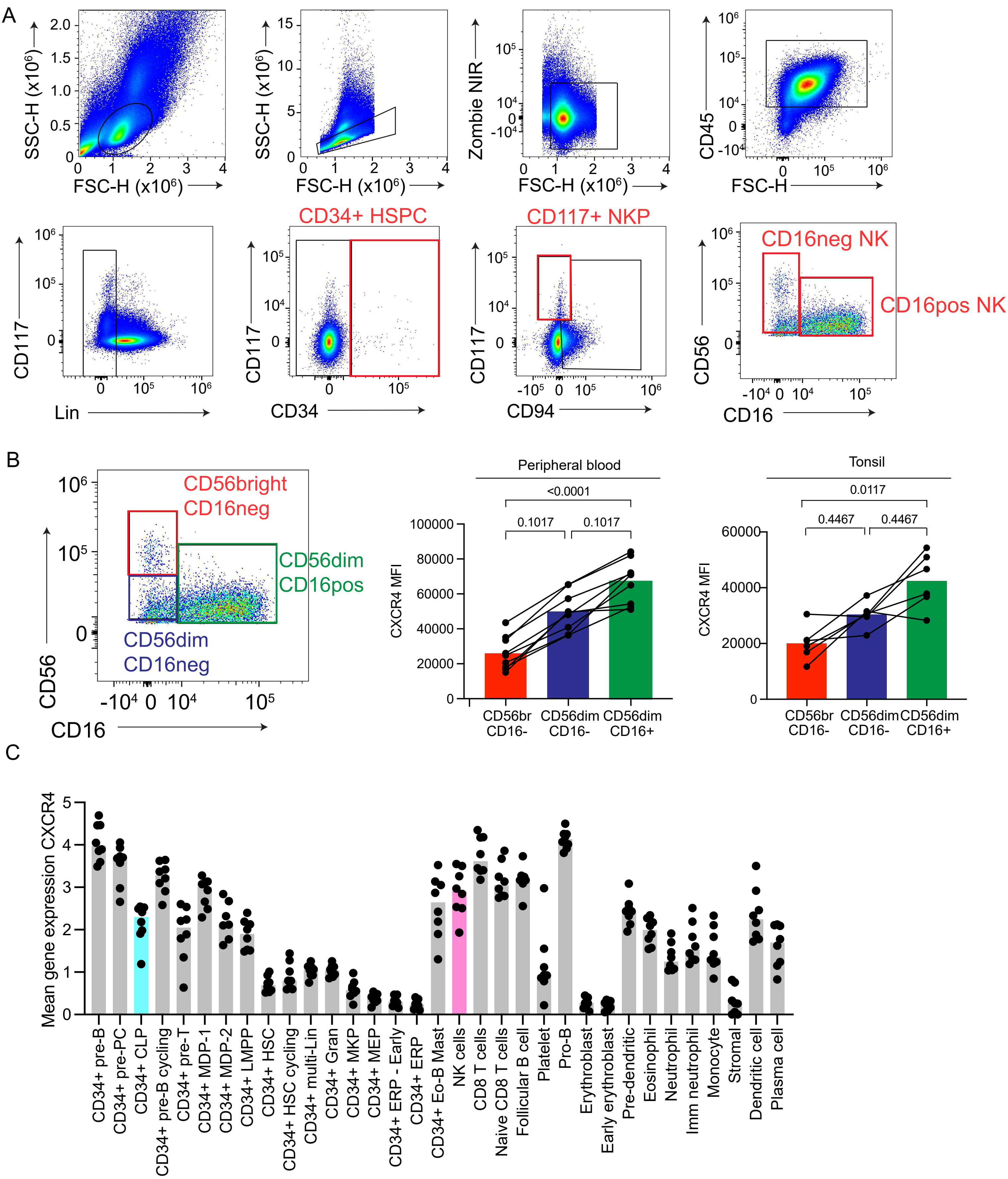

Supplemental Figure 1. Gating strategy and CXCR4 expression. A) Gating strategy used for analysis of NK cell developmental intermediates in peripheral blood and tonsil. B) Additional gating of mature NK cell subsets from peripheral blood and tonsil (left). Quantitation of CXCR4 MFI from peripheral blood and tonsil mature NK cell subsets. n=9 (peripheral blood) and 6 (tonsil) donors; p-values generated by Friedman test. C) Mean gene expression of *CXCR4* from Hay et al. (ref. 40) highlighting CD34+ common lymphoid progenitors (cyan) and NK cells (pink). Mean counts per 10,000 cells normalized.

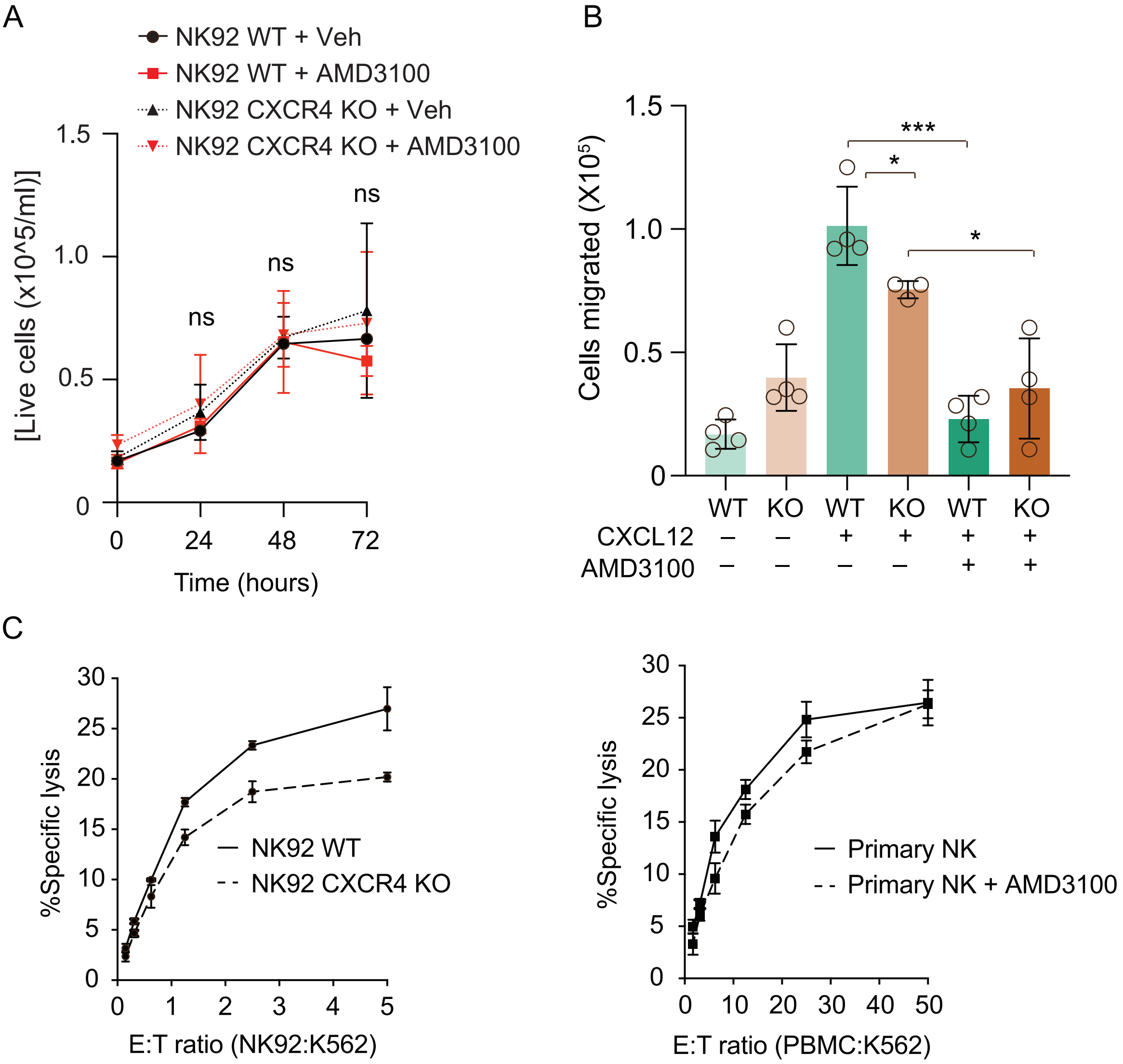

Supplemental Figure 2. Proliferation assays, transwell assays, and killing assays A) NK92 WT and CXCR4 KO cell proliferation measured over 72h in the presence of Vehicle (water) or 10μM AMD3100. Lines show mean±SD (n=3 independent experiments). ns=not significant by two-way ANOVA. B) CXCL12-directed chemotaxis of NK92 WT and CXCR4 KO cells ±AMD3100 in transwell migration assays; mean±SD (n=3 independent experiments). P values determined by Mann-Whitney test between samples as indicated. C) Chromium cytotoxicity assays using NK92 (left) or peripheral blood mononuclear cells (right) co-cultured with K562 target cells at indicated effector: target (E:T) ratios. Mean±SEM.

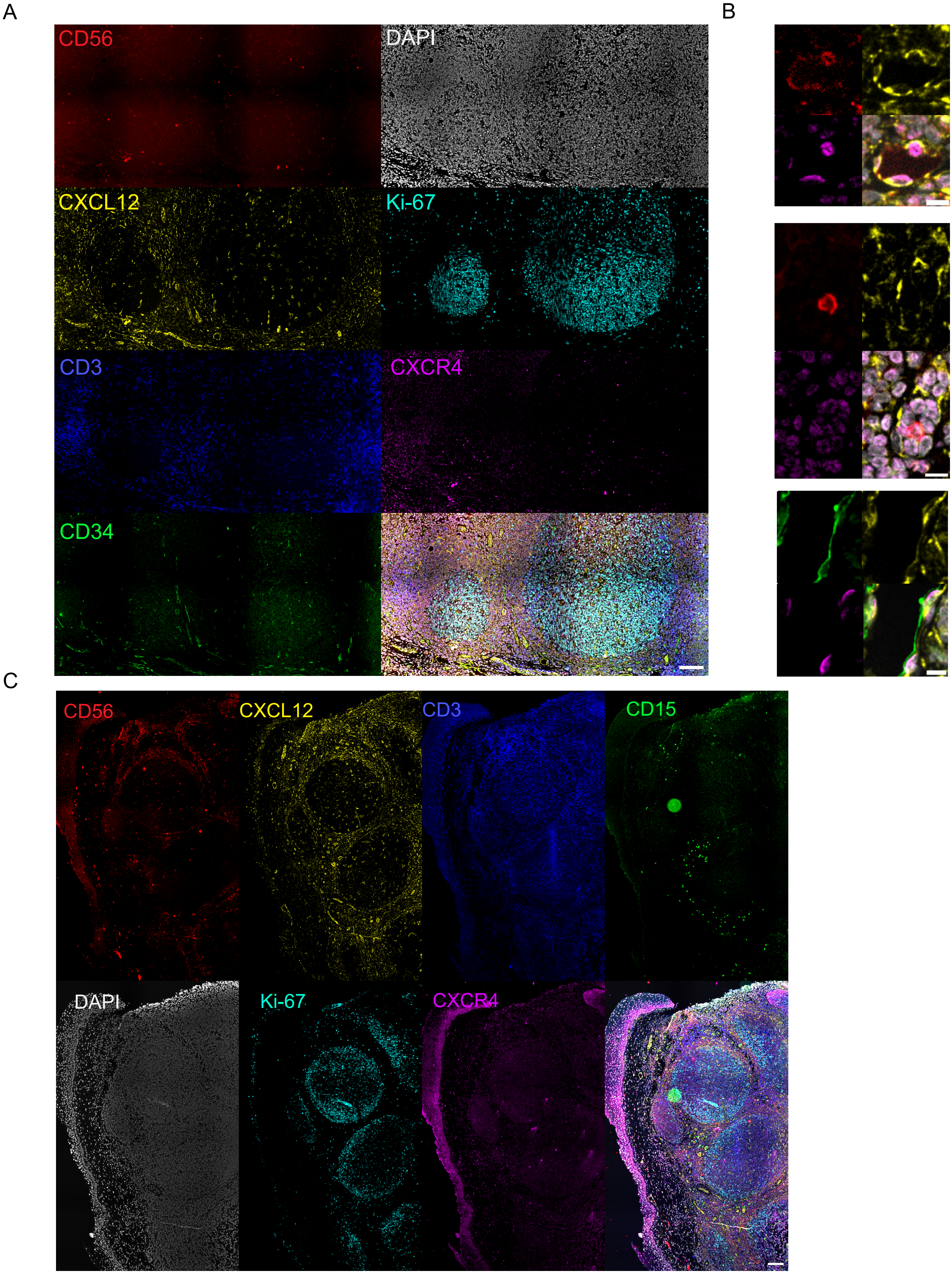

Supplemental Figure 3. Cyclic immunofluorescence (CyCIF) imaging of pediatric tonsil identifying CXCL12-rich stromal regions and associated immune populations. A) Representative CyCIF panel from pediatric tonsil tissue showing individual channels for CD56 (red), DAPI (gray), CXCL12 (yellow), Ki-67 (cyan), CD3 (blue), CXCR4 (magenta), and CD34 (green). B) Insights highlight CXCR4+ CD56+ and CXCR4+ CD34+ cells positioned near CXCL12-producing stromal structures. C) Second representative region with individual channels for CD56 (red), DAPI (gray), CXCL12 (yellow), Ki-67 (cyan), CD3 (blue), CXCR4 (magenta), and CD15 (green).

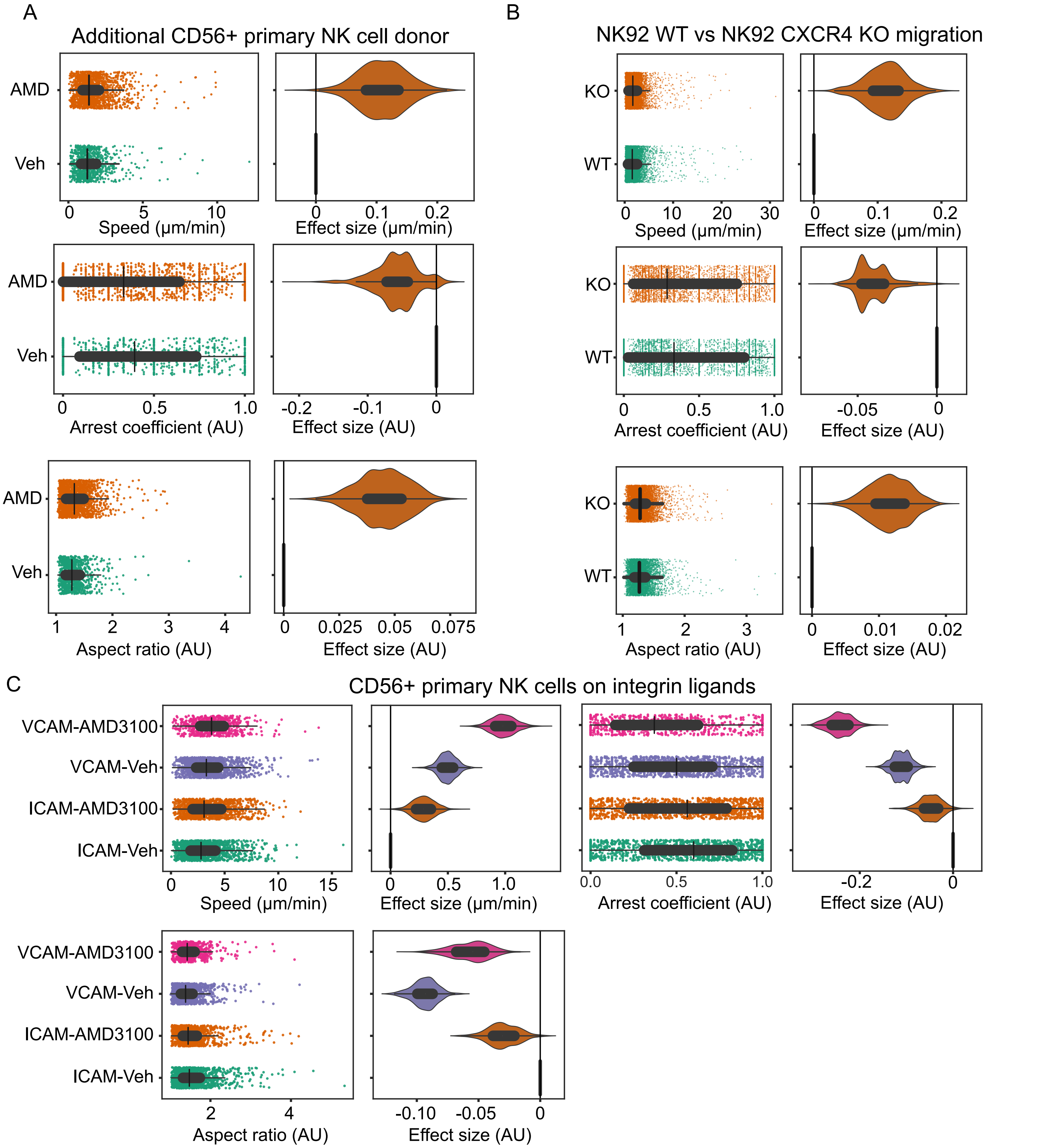

Supplemental Figure 4. Plots of migration and morphological metrics from additional experimental replicates. A) NK cells were isolated from peripheral blood of healthy donors and incubated on EL08.1D2 stromal cells in the presence of AMD3100 or Vehicle as indicated then imaged by confocal microscopy for 4 hours at 2 min intervals. Point plots display pooled single-cell metrics with effect size distributions. B) NK92 WT or CXCR4-KO cells were incubated on EL08.1D2 stromal cells then imaged by confocal microscopy for 4 hours at 2 min intervals. Point plots display pooled single-cell metrics with effect size distributions. C) NK cells were isolated from peripheral blood of healthy donors and incubated on recombinant VCAM1 or ICAM1 in the presence of AMD3100 or Vehicle as indicated then imaged by confocal microscopy for 4 hours at 2 min intervals. Point plots display pooled single-cell metrics with effect size distributions.

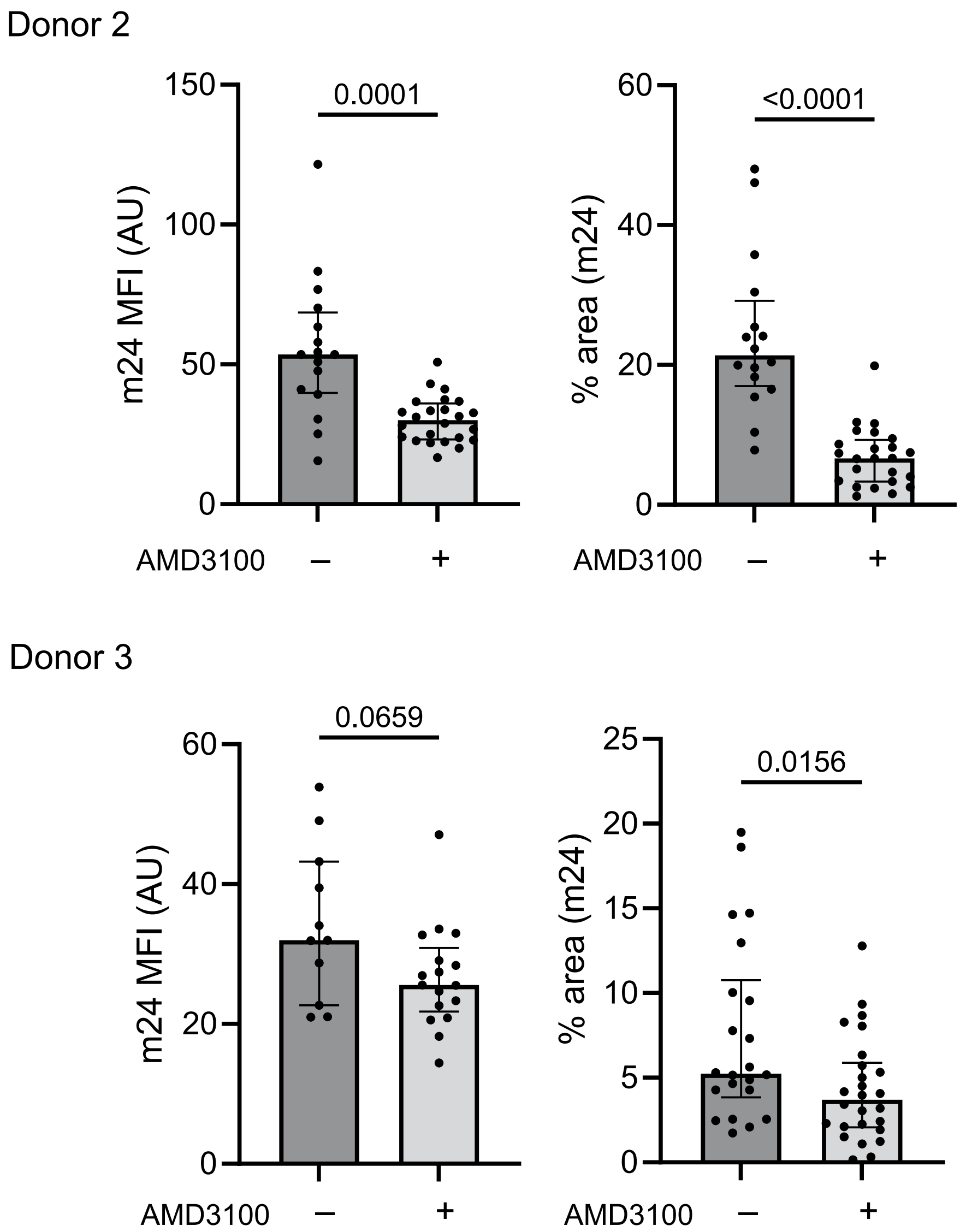

Supplemental Figure 5. Additional donors for m24 MFI and percent area. Quantification of m24 MFI and percent area from two additional healthy donors (see Fig. 4E for Donor 1). Each dot represents a single cell from one donor. Horizontal bars indicate mean±SD. Statistical comparison was performed using a two-tailed Mann-Whitney test.

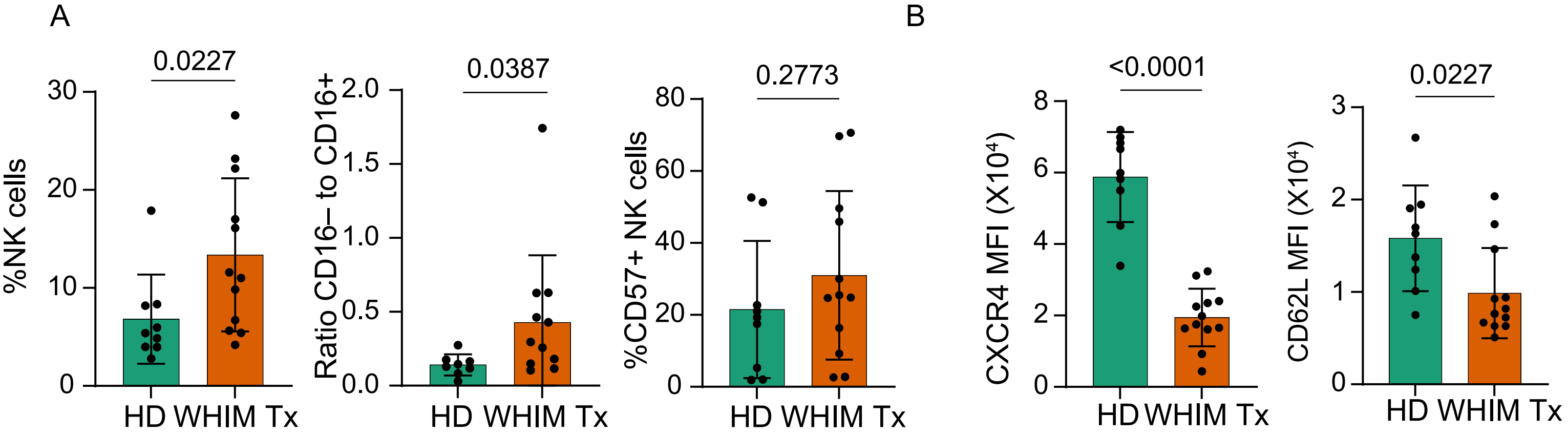

Supplemental Figure 6. NK cell numbers from WHIM syndrome patients. A) NK cell frequency, CD16+ and CD57+ subsets in healthy donors (HDs) or WHIM patients treated with filgrastim or plerixafor (WHIM Tx). n=9 HD samples and 12 WHIM patient samples; mean±SD; Mann-Whitney test. B) CXCR4 and CD62L on NK cells from HD and treated WHIM patients (WHIM Tx); mean±SD; Mann-Whitney test.

Supplemental Table 1: Antibody panel used for flow cytometric analysis of NK cell developmental subsets

Antigen	Clone	Fluorochrome	Vendor	Catalog #	Dilution
**CD45**	HI30	BUV805	BD Biosciences	612891	1:100
**CD94**	HP-3D9	BUV395	BD Biosciences	743954	1:150
**CD3**	UCHT1	BUV496	BD Biosciences	612940	1:200
**CD14**	M5E2	BUV496	BD Biosciences	750381	1:200
**CD19**	SJ25C1	BUV496	BD Biosciences	612938	1:200
**CD56**	NCAM16.2	BUV563	BD Biosciences	612928	1:100
**CD57**	QA17AO4	BV510	Biolegend	393314	1:100
**CD117**	104D2	BV711	Biolegend	313230	1:100
**CD34**	561	PE Dazzle	Biolegend	343534	1:100
**CXCR4**	12G5	PE-Cy7	Biolegend	306514	1:100
**NKp80**	REA845	APC	Miltenyi	130-112-591	1:100
**CD16**	3G8	BV650	Biolegend	302042	1:150
**CD103**	Ber-ACT8	BV785	Biolegend	350230	1:150
**CD49a**	TS2/7	FITC	Biolegend	328308	1:100
**CCR7**	G043H7	BV421	Biolegend	353208	1:100
**CD69**	FN50	PE	Biolegend	310906	1:100
**CD62L**	DREG-56	AF700	Biolegend	304820	1:100

**Eomes**	WD1928	APC	Invitrogen	50-4877-42	1:100
**Granzyme B**	GB11	BV421	Biolegend	515408	1:100

Supplemental Table 1: Antibody panel used for cyclic immunofluorescence of FFPE pediatric tonsil sections

Antigen	Clone	Vendor	Catalog #	Conc (ug/mL)
**Ki67**	B56	Fluidigm	3172024B	5
**CXCL12**	79018	RnD	MAB350-SP	8
**CD3**	Polyclonal	abcam	AB5690	1.5
**CD56**	Polyclonal	RnD	AF2408	2.5
**CXCR4**	UMB2	abcam	ab124824	2
**CD34**	QBEnd/10	Novus	NBP2-34713	5

Supplemental Table 2: Quantification of migration parameters for migration plots shown and for additional biological donors.

CD56+ NK cells (Donor 1) + vehicle or AMD3100 on EL08.1D2 (Fig. 3C)							
**metric**	**measurement type (per frame or per track)**	**units**	**Vehicle_mean**	**Vehicle_N**	**AMD_Mean**	**AMD_N**	**p AMD vs Vehicle**
speed	per frame	µm/min	0.9857	10994	1.2336	27370	4.3209E-27
arrest coefficient	per track	fraction	0.4830	2271	0.3740	6204	1.6277E-32
aspect ratio	per frame	unitless	1.3185	10994	1.3123	27370	8.8468E-07
**CD56+ NK cells (Donor 2) + vehicle or AMD3100 on EL08.1D2 (Supp. Fig. 4A)**							
**metric**	**measurement type (per frame or per track)**	**units**	**Vehicle_mean**	**Vehicle_N**	**AMD_Mean**	**AMD_N**	**p AMD vs Vehicle**
speed	per frame	µm/min	1.0969	8123	1.2407	13363	5.2739E-04
arrest coefficient	per track	fraction	0.4227	856	0.3741	1715	4.2769E-04
aspect ratio	per frame	unitless	1.2456	8123	1.2873	13363	3.7699E-04
**CD34+ HSPCs (Donor 1) + vehicle or AMD3100 on EL08.1D2 (Fig. 3D)**							
**metric**	**measurement type (per frame or per track)**	**units**	**Vehicle_mean**	**Vehicle_N**	**AMD_Mean**	**AMD_N**	**p AMD vs Vehicle**
speed	per frame	µm/min	0.8266	7206	0.8816	22568	5.1072E-02
arrest coefficient	per track	fraction	0.5367	1245	0.5099	3944	1.7822E-02
aspect ratio	per frame	unitless	1.4078	7206	1.3139	22568	1.3626E-18
**NK92 WT vs NK92 CXCR4 KO (Replicate 1) on EL08.1D2 (Supp. Fig. 4B)**							
**metric**	**measurement type (per frame or per track)**	**units**	**WT_mean**	**WT_N**	**KO_Mean**	**KO_N**	**p KO vs WT**
speed	per frame	µm/min	1.6312	5267	1.6720	6757	4.7481E-01
arrest coefficient	per track	fraction	0.4322	48737	0.4039	73563	3.3858E-05
aspect ratio	per cell	unitless	1.2601	5267	1.2708	6757	5.3127E-03
**NK92 WT vs NK92 CXCR4 KO (Replicate 2) on EL08.1D2 (Plots not shown)**							
**metric**	**measurement type (per frame or per track)**	**units**	**WT_mean**	**WT_N**	**KO_Mean**	**KO_N**	**p KO vs WT**
speed	per frame	µm/min	1.2012	513889	1.5097	299843	0.0000
arrest coefficient	per track	fraction	0.5306	27973	0.4130	16413	4.8415E-50
aspect ratio	per cell	unitless	1.2194	513889	1.2409	299843	0.0000
**CD56+ NK cells (Donor 1) + vehicle or AMD3100 on ICAM surface (Sup. Fig. 4C)**							
**metric**	**measurement type (per frame or per track)**	**units**	**Vehicle_mean**	**Vehicle_N**	**AMD_Mean**	**AMD_N**	**p AMD vs Vehicle**
speed	per frame	µm/min	2.5538	25619	2.8919	26903	1.5578E-04
arrest coefficient	per track	fraction	0.5585	1322	0.5165	1165	1.0279E-03
aspect ratio	per frame	unitless	1.5907	25619	1.5782	26903	3.4494E-03
**CD56+ NK cells (Donor 2) + vehicle or AMD3100 on ICAM surface (Plots not shown)**							

metric	measurement type (per frame or per track)	units	Vehicle_mean	Vehicle_N	AMD_Mean	AMD_N	p AMD vs Vehicle
speed	per frame	µm/min	2.7770	54093	3.0310	46307	2.2940E-04
arrest coefficient	per track	fraction	0.2835	3268	0.2616	2409	7.5613E-03
aspect ratio	per frame	unitless	1.4740	54093	1.4959	46307	6.0714E-01
**CD56+ NK cells (Donor 1) + vehicle or AMD3100 on VCAM surface (Supp. Fig. 4C)**							
**metric**	**measurement type (per frame or per track)**	**units**	**Vehicle_mean**	**Vehicle_N**	**AMD_Mean**	**AMD_N**	**p AMD vs Vehicle**
speed	per frame	µm/min	3.1107	17919	3.1657	10507	2.2426E-05
arrest coefficient	per track	fraction	0.4863	1423	0.4055	698	2.8182E-09
aspect ratio	per frame	unitless	1.4312	17919	1.4815	10507	3.5592E-04
**CD56+ NK cells (Donor 2) + vehicle or AMD3100 on VCAM surface (Plot not shown)**							
**metric**	**measurement type (per frame or per track)**	**units**	**Vehicle_mean**	**Vehicle_N**	**AMD_Mean**	**AMD_N**	**p AMD vs Vehicle**
speed	per frame	µm/min	0.9367	51977	1.9258	16393	4.8629E-14
arrest coefficient	per track	fraction	0.6560	433	0.4175	154	8.1200E-14
aspect ratio	per frame	unitless	1.4502	51977	1.3194	16393	2.9630E-02
**CD56+ NK cells from WHIM patient (Donor #7) after plerixafor or filgrastim treatment on EL08.1D2 (Fig. 6D)**							
**metric**	**measurement type (per frame or per track)**	**units**	**Filgrastim_mean**	**Filgrastim_N**	**Plerixafor_Mean**	**Plerixafor_N**	**p plerix vs filgr**
speed	per frame	µm/min	0.6777	15444	0.8724	44817	5.0241E-09
arrest coefficient	per track	fraction	0.5125	2006	0.4489	7394	9.7301E-13
aspect ratio	per frame	unitless	1.2608	15444	1.1886	44817	7.5587E-63
**CD56+ NK cells from WHIM patient (Donor #1) after plerixafor or filgrastim treatment on EL08.1D2 (Plots not shown)**							
**metric**	**measurement type (per frame or per track)**	**units**	**Filgrastim_mean**	**Filgrastim_N**	**Plerixafor_Mean**	**Plerixafor_N**	**p plerixafor vs filgrastim**
speed	per frame	µm/min	0.5542	11295	0.5565	2307	3.8647E-04
arrest coefficient	per track	fraction	0.5689	1265	0.4818	189	7.8480E-03
aspect ratio	per frame	unitless	1.1959	11295	1.1183	2307	9.8265E-10
